# Spectral and Directed Connectivity EEG Markers for Classifying Alzheimer’s Disease, Frontotemporal Dementia, and Healthy Controls with Exploratory Photobiomodulation Case-Study Projection

**DOI:** 10.3390/s26175530

**Published:** 2026-08-31

**Authors:** Zoran Šverko, Saša Vlahinić, Miroslav Vrankić, Nino Stojković

**Affiliations:** 1 Department of Electric Power Systems, Faculty of Engineering, University of Rijeka, 51000 Rijeka, Croatia; 2 Department of Automation and Electronics, Faculty of Engineering, University of Rijeka, 51000 Rijeka, Croatia

**Keywords:** Alzheimer’s disease, frontotemporal dementia, electroencephalography, Granger causality, directed functional connectivity, spectral analysis, theta-to-alpha ratio, photobiomodulation

## Abstract

Alzheimer’s disease (AD) and frontotemporal dementia (FTD) are neurodegenerative disorders with partially overlapping clinical manifestations, making early and differential diagnosis challenging. This study investigated whether electroencephalography (EEG)-derived spectral features and Granger-causality (GC)-based directed functional connectivity features can characterize and classify AD, FTD, and healthy control (HC) subjects. Resting-state eyes-closed EEG recordings from 88 participants were analyzed, including 36 AD, 23 FTD, and 29 HC subjects. Spectral features included absolute and relative band power and spectral ratios, while directed connectivity features were extracted from broadband and frequency-specific GC matrices. Statistical analyses identified theta/alpha ratio (TAR) as the dominant spectral marker, with the strongest three-group differences observed in frontal and global TAR features. GC analysis revealed group-related alterations mainly in alpha-band regional directed connectivity, although three-group GC features did not survive false discovery rate (FDR) correction at q < 0.05. In the main nested cross-validation analysis, the spectral-only model achieved the best three-class performance, with balanced accuracy of 0.572 and macro-F1 of 0.557. For dementia group (DEM) vs. HC classification, the combined GC + spectral feature (GC + SPEC) set achieved balanced accuracy of 0.710 and macro-F1 of 0.665. For AD vs. FTD classification, the combined GC + SPEC feature set achieved the highest numerical performance in the main nested cross-validation (CV) comparison, with balanced accuracy of 0.584 and macro-F1 of 0.559. In the separate long permutation-testing analysis, which used a reduced hyperparameter grid for computational feasibility, above-chance performance was confirmed for the three-class spectral model and the DEM vs. HC GC + SPEC model (*p* < 0.001), but not for AD vs. FTD (*p* = 0.270). An exploratory photobiomodulation (PBM) single-case analysis showed longitudinal EEG reorganization, including increased alpha power, reduced delta/alpha ratio (DAR) and beta/alpha ratio (BAR), mixed TAR changes, and HC-like GC/GC + SPEC centroid projections. Overall, the results support the value of spectral and directed connectivity EEG markers for dementia-related EEG characterization, while highlighting the persistent difficulty of AD vs. FTD differentiation.

## 1. Introduction

AD and FTD are progressive neurodegenerative disorders with partially overlapping clinical manifestations, which can complicate early and differential diagnosis. AD is the most common cause of dementia and is typically associated with progressive memory impairment and broader cognitive decline, whereas FTD is more commonly characterized by early changes in behavior, personality, executive function, and language. Despite these differences, symptom overlap between dementia subtypes, especially in earlier disease stages, may lead to diagnostic uncertainty and delayed differentiation between AD, FTD, and cognitively healthy individuals [1,2].

Current diagnostic procedures rely on clinical assessment, neuropsychological testing, neuroimaging, and, when available, fluid or molecular biomarkers. Magnetic resonance imaging (MRI), positron emission tomography (PET), and cerebrospinal fluid biomarkers provide important structural, metabolic, and molecular information, but they can be expensive, invasive, or less accessible in routine clinical settings. Consequently, there is growing interest in complementary electrophysiological approaches that are non-invasive, cost-effective, and suitable for repeated measurements. EEG is particularly relevant in this context because it directly records brain electrical activity with high temporal resolution and can capture alterations in neural oscillations and functional network organization associated with neurodegenerative disease [3,4].

Resting-state EEG studies in dementia have consistently reported changes in spectral activity. In AD, a characteristic shift toward slower oscillatory activity is commonly observed, including increased delta and theta activity together with reduced alpha and beta activity. Spectral ratios, especially the TAR, have been used to quantify this slowing and have been reported as discriminative markers between AD and HC [2,5,6]. FTD-related EEG findings are less consistent across studies, but several reports suggest that FTD may involve distinct spectral and spatial patterns, particularly within frontal, temporal, and central regions [2,4,7]. These observations support the use of spectral EEG features for characterizing dementia-related changes, while also indicating that spectral measures alone may not fully capture the complexity of disease-specific neural dysfunction.

In addition to spectral analysis, EEG functional connectivity has become an important framework for studying network-level alterations in dementia. Functional connectivity measures quantify statistical dependencies between signals recorded at different scalp locations and can provide insight into the organization of large-scale brain communication. Previous studies have used measures such as coherence, phase lag index, weighted phase lag index, mutual information, graph-theoretical metrics, and minimum spanning tree analysis to characterize AD- and FTD-related network abnormalities [4,7,8,9]. These approaches have shown that dementia is not only associated with local oscillatory changes, but also with alterations in distributed brain networks. However, many commonly used connectivity measures are undirected and therefore do not provide information about the direction of information flow between brain regions.

Directed functional connectivity may provide additional information by estimating whether activity in one EEG channel contributes to the prediction of activity in another channel. GC is one such approach and has been widely used to quantify predictive directional interactions between time series. In EEG analysis, GC can be used to estimate directed interactions between electrode pairs across frequency bands, thereby allowing investigation of source-to-target relationships within functional brain networks. This is particularly relevant for AD and FTD because both conditions are associated with altered network organization, while FTD prominently affects frontal and temporal systems and AD is often associated with posterior and distributed network disruption [4,7,8].

Recent machine learning studies using EEG data have demonstrated the potential of automated approaches for distinguishing AD, FTD, and HC. Several studies have evaluated spectral, time-domain, nonlinear, and connectivity-based EEG features, with classification performance varying depending on feature extraction, validation strategy, and classifier design [2,5,7,8,10,11,12,13]. Although high classification accuracies have been reported in some studies, recent work also emphasizes the importance of subject-level validation, careful feature engineering, and transparent evaluation procedures in small EEG datasets [8]. In particular, distinguishing dementia subjects from healthy controls is generally more robust than differentiating AD from FTD, which remains a clinically and computationally challenging task because of overlapping neurophysiological alterations [2,7,8]. A structured comparison of selected previous EEG-based AD/FTD/HC studies and their methodological limitations relative to the present GC-based approach is provided in the Discussion.

EEG-derived spectral and connectivity markers are also relevant beyond diagnostic classification, particularly for longitudinal monitoring of neurophysiological changes. Photobiomodulation (PBM), a non-invasive intervention based on red or near-infrared light stimulation, has been investigated in dementia and cognitive impairment, with previous EEG-based work reporting changes [6,14] in theta and alpha activity, TAR, and connectivity-related measures after stimulation. Although PBM intervention is not the primary focus of the present study, these findings further support the relevance of EEG spectral ratios and connectivity measures as quantitative markers for monitoring neurophysiological alterations over time.

Building on these considerations, the present study investigates resting-state EEG features for the characterization and classification of AD, FTD, and HC subjects. Spectral features, including absolute and relative band power and spectral ratios such as TAR, DAR, and BAR, were extracted together with GC-derived directed connectivity features computed across broadband and frequency-specific EEG signals. Exploratory statistical analyses were performed to describe group-related EEG feature differences, while nested cross-validation was used to evaluate classification performance for three-class AD/FTD/HC classification and for binary DEM vs. HC and AD vs. FTD tasks. Finally, permutation testing was applied to selected classification models to assess whether their performance exceeded chance level.

The novelty of the present study lies in the integrated evaluation of complementary EEG marker families within the same AD/FTD/HC analysis framework. Rather than evaluating spectral slowing or connectivity features in isolation, this study combines spectral power, spectral ratios, broadband and frequency-specific GC-derived directed connectivity descriptors, subject-level nested cross-validation, and permutation testing. This design allows the relative contribution of spectral and directed connectivity features to be compared across three clinically relevant classification tasks: three-class AD/HC/FTD classification, DEM vs. HC classification, and AD vs. FTD differential classification. In addition, the exploratory PBM case-study projection extends the analysis from cross-sectional group classification toward longitudinal EEG-marker monitoring, while remaining descriptive and non-diagnostic.

The specific unresolved problem addressed by this study is therefore whether spectral slowing markers and GC-derived directed connectivity descriptors provide complementary and statistically supported information for dementia-related EEG characterization when evaluated under the same subject-level validation framework. We hypothesized that TAR-based spectral features would provide the most robust group-level information, particularly for separating dementia subjects from HC, whereas GC-derived directed connectivity descriptors would provide complementary network-level information reflecting regional source-to-target interactions and could improve selected binary classification tasks. We further expected AD vs. FTD differentiation to remain more challenging than DEM vs. HC classification because of overlapping neurophysiological alterations between dementia subtypes.

## 2. Materials and Methods

**Figure 1:**
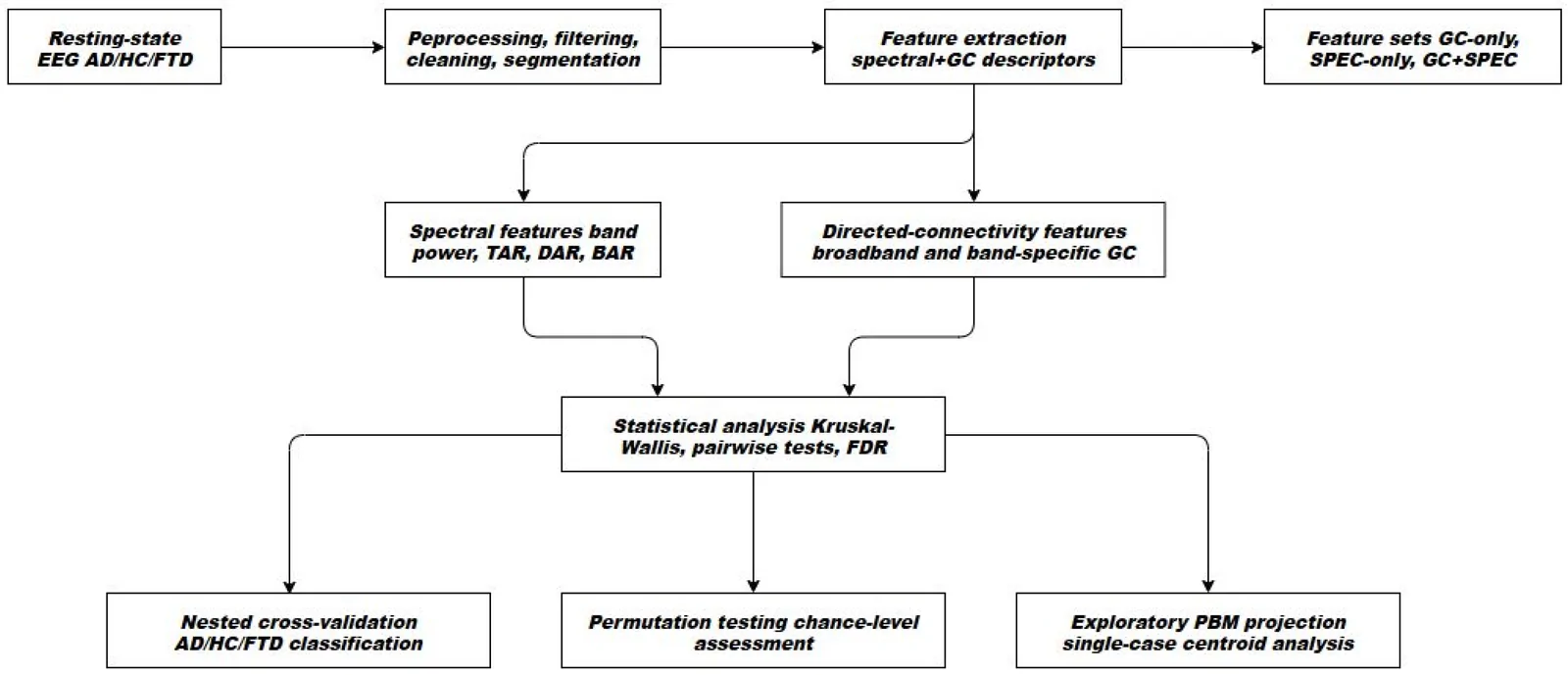
Overview of the complete EEG analysis and classification workflow.

The complete processing and analysis workflow is summarized in Figure 1. The pipeline included EEG data input, preprocessing, spectral-feature extraction, GC-based directed connectivity feature extraction, statistical analysis, classification with nested cross-validation, permutation testing, and exploratory PBM case-study projection.

### 2.1. EEG Dataset

The EEG data used in this study were obtained from a publicly available resting-state EEG dataset of subjects with AD, FTD, and HC, originally collected at the 2nd Department of Neurology, AHEPA General Hospital, Thessaloniki, Greece, and described by Ref. [15]. This dataset consists of raw resting-state, eyes-closed EEG recordings from a total of 88 participants, including 36 subjects diagnosed with AD, 23 subjects diagnosed with FTD, and 29 cognitively healthy (HC) subjects. EEG signals were recorded using a Nihon Kohden EEG 2100 system with 19 scalp electrodes positioned according to the international 10–20 system. During the recordings, the participants were seated in a resting state with their eyes closed. The sampling frequency was 500 Hz.

### 2.2. EEG Signal Import, Preprocessing, and Frequency Sub-Band Extraction

Raw EEG recordings were imported into MATLAB R2010b using the EEGLAB toolbox. Channel locations were assigned according to the 19-channel 10–20 montage, and the signals were re-referenced to the average reference. This initial average referencing step was applied to reduce dependence on the original recording reference and to place all channels in a common reference framework before filtering, noisy-channel screening, and ICA-based artifact identification. Subsequently, a 0.5–45 Hz band-pass filter was applied in order to retain only the frequency range relevant for further spectral analysis and functional connectivity analysis. Detection of channels producing noisy signals was performed using a spectral channel rejection procedure with a z-score threshold of 5, while independent components associated with artifacts were identified using the ICLabel algorithm [16]. ICLabel was used as an automated probabilistic classifier of ICA components, whereas the rejection thresholds applied in this study were selected by the authors as conservative operational criteria for artifact-component removal. Specifically, if the probability that a component represented brain activity was ≤0.05, or if the probability of belonging to any artifact class was ≥0.90, the component was removed. This ICA/ICLabel-based step was particularly relevant for reducing ocular contamination, because frontal electrodes are more susceptible to eye-blink and eye-movement activity. Components classified as artifact-related, including eye-related components when detected by ICLabel, were removed before spectral and GC-derived feature extraction. However, no separate EOG channels were available in the present analysis; therefore, residual ocular activity, especially in frontal electrodes, cannot be completely excluded. After artifact removal, the reconstructed cleaned EEG signals were again re-referenced to the average reference, because ICA component rejection changes the composition of the sensor-level signals. The processed signals were then divided into standard frequency sub-bands: delta (0.5–4 Hz), theta (4–8 Hz), alpha (8–13 Hz), low beta (13–18 Hz), high beta (18–30 Hz), and gamma (35–45 Hz). The resulting band-limited signals were used for subsequent spectral feature extraction and GC-based directed connectivity analysis.

### 2.3. Spectral Feature Extraction

Spectral features were extracted from the preprocessed broadband signal and the corresponding sub-bands. The power of each frequency band was calculated as the mean squared amplitude of the corresponding band-limited signal for each subject and each electrode. Absolute power was calculated for the delta, theta, alpha, low beta, high beta, gamma, and total beta ranges. Furthermore, relative power was calculated by normalizing the power of each frequency band by the broadband EEG power. Features were calculated at three levels: first, at the level of individual electrodes; second, global features were obtained by averaging across all 19 electrodes; and third, regional features were calculated for predefined anatomical regions, including frontal, central, temporal, parietal, and occipital regions.

Several spectral ratios were also calculated to quantify the relative contribution of slower and faster EEG rhythms. The TAR was defined as the ratio between theta-band power and alpha-band power, the DAR was calculated as the ratio between delta-band power and alpha-band power, and the BAR was calculated as the ratio between total beta power and alpha-band power. These ratios were also calculated at three levels, namely at the electrode, regional, and global levels. The TAR measure was included because theta and alpha activity, and particularly their ratio, have previously been used as quantitative EEG markers [6] in cognitive impairment and in the analysis of EEG changes associated with photobiomodulation [6,14].

### 2.4. Granger-Causality-Based Directed Connectivity Analysis

Directed functional connectivity in this study was estimated using GC. GC is a statistical approach used to evaluate directed dependencies between two time series. In this analysis, a signal (S), referred to as the source signal, was considered to Granger-cause another signal (T), referred to as the target signal. If the past values of signal (S) improved the prediction of the current value of signal (T), compared with a prediction based exclusively on the past values of signal (T), then GC was considered to be present. Consequently, GC does not imply direct anatomical or physical causation, but rather predictive causality based on temporal information contained in the signals.

The target signal (T(n)) was modeled using a univariate autoregressive (AR) model, in which the current value of the signal was predicted from its previous samples:

(1) $$T\left(n\right)=\sum_{i=1}^{M}\omega_{T_{i}}T\left(n-i\right)+e_{T}\left(n\right),$$

where (T(n)) denotes the current sample of the target EEG signal, (*M*) is the autoregressive model order, ($\omega_{T_{i}}$) are the AR coefficients associated with the previous samples of the target signal, and ($e_{T}\left(n\right)$) is the prediction error of the univariate model. From this perspective, this model estimates how well the future value of the target signal can be predicted using only its own past activity.

Furthermore, to examine whether the source signal (S(n)) provided additional predictive information, a bivariate autoregressive model was used:

(2) $$\begin{array}{cc} T(n) & =\sum_{i=1}^{M}\omega_{T_{i}}T\left(n-i\right)\\ \; & +\sum_{i=1}^{M}\omega_{S_{i}}S\left(n-i\right)+e_{TS}\left(n\right). \end{array}$$

where (S(n)) denotes the source EEG signal, ($\omega_{S_{i}}$) are the model coefficients associated with the previous samples of the source signal, and ($e_{TS}\left(n\right)$) is the prediction error after including the source signal in the model. If the inclusion of the past values of (S(n)) reduced the prediction error of (T(n)), then a directed influence from signal (S) to signal (T) was inferred.

The GC value was calculated as the ratio between the prediction error variance of the univariate model and the prediction error variance of the bivariate model, according to the following relation:

(3) $$GC_{S\to T}=\frac{var[e_{T}(n)]}{var[e_{TS}(n)]}.$$

A higher GC value indicates that including the history of the source signal during prediction more strongly reduced the prediction error of the target signal, corresponding to a stronger directed influence from (S) to (T). In this study, the autoregressive model order was set to (M = 19), with the model order selected using the Akaike information criterion (AIC). Here, M denotes the number of temporal lags included in the AR model and not the number of EEG electrodes. Therefore, the equality between M = 19 and the 19 scalp electrodes used in the dataset is coincidental and has no methodological relationship to the number of channels. To improve the stability of high-order AR model estimation, GC was calculated over the entire cleaned EEG recording.

For each subject and each investigated frequency band, GC values were calculated between all pairs of electrodes, resulting in a (19 × 19) directed connectivity matrix. For each electrode pair ((i,j)), the matrix element (GC(i,j)) represented the causal influence of electrode (j) on electrode (i). Therefore, the rows of the matrix represented target electrodes, whereas the columns represented source electrodes. Diagonal elements, corresponding to self-connections, were excluded from further analysis. This procedure was repeated for the broadband signal and for each frequency sub-band: delta, theta, alpha, low beta, high beta, and gamma.

It should be noted that the GC analysis in this study was performed in a bivariate pairwise manner. Therefore, each source-target electrode pair was evaluated independently of the remaining electrodes. This approach does not explicitly control for the influence of other simultaneously recorded channels and may be affected by common-source activity, volume conduction, or indirect interactions. Consequently, the GC values reported here should be interpreted as sensor-level predictive directed connectivity markers rather than as evidence of direct anatomical or physical causal pathways.

Furthermore, several directed connectivity features were extracted from each GC matrix. Global features described the overall distribution of GC values across all electrode pairs. Node-level features included incoming strength, outgoing strength, and net directed flow for each electrode, where incoming strength was calculated by summing incoming influences toward the target electrode, outgoing strength by summing outgoing influences from the source electrode, and net flow as the difference between outgoing and incoming strength. In addition, regional source-region-to-target-region features were calculated by averaging GC values between predefined scalp-electrode groupings based on the 10–20 montage: frontal, central, temporal, parietal, and occipital. These labels refer to scalp-level electrode groupings and should not be interpreted as reconstructed cortical source regions. The resulting features were used to describe the strength and direction of information flow in the EEG sensor network.

### 2.5. Statistical Analysis

Exploratory statistical analysis was performed separately for the spectral feature set and for the feature set derived from the GC analysis. Since the extracted EEG features included power ratios and connectivity measures, their distributions were not assumed to be normally distributed. Therefore, non-parametric statistical tests were used. For comparisons among all three groups of AD, FTD, and HC subjects, the Kruskal–Wallis test [2] was applied independently to each feature. This test was selected because it allows comparison of more than two independent groups without assuming normally distributed data. The nominal significance level was set to (α=0.05).

To further examine pairwise differences between groups, Wilcoxon rank-sum tests [2] were performed for the AD vs. HC, HC vs. FTD, and AD vs. FTD comparisons. These pairwise tests were applied only as exploratory follow-up analyses in order to identify which group pairs contributed to the observed three-group differences. In addition, a binary exploratory analysis was performed in which AD and FTD subjects were combined into a DEM, which was then compared with HC subjects. This additional comparison was included to evaluate whether the extracted EEG features captured a broader dementia-related pattern, independently of dementia subtype.

For analyses involving multiple features, *p*-values were corrected for multiple comparisons using the Benjamini–Hochberg FDR procedure [7]. FDR correction was applied separately within each family of tests, including the three-group feature analysis, the pairwise group comparisons, and the DEM vs. HC analysis. The FDR significance threshold was set to (q < 0.05). For each feature, group-specific means and medians were also calculated. Means were used to describe the overall magnitude and direction of group differences, whereas medians were reported because they are more robust to skewed distributions and extreme values. The exploratory statistical analyses were used to describe EEG feature patterns associated with the diagnostic groups and to support interpretation of the results. They were not used for feature selection in the main classification analyses, in order to avoid information leakage from the test folds into the model training procedure.

### 2.6. Classification Procedure

#### 2.6.1. Feature Sets and Classification Tasks

Classification analyses were performed to evaluate whether the extracted EEG features could distinguish between AD, FTD, and HC subjects. Three feature sets were evaluated: features derived only from GC analysis, features derived only from spectral analysis, and a combined feature set containing both GC features and spectral features. The GC-only feature set contained global and regional directed connectivity features extracted from the GC matrices. The spectral feature set contained absolute band power, relative band power, and spectral ratios calculated at the electrode, regional, and global levels. The combined feature set was obtained by combining the GC and spectral feature matrices.

The main classification task was a three-class classification problem involving AD, FTD, and HC subjects. In addition, two binary classification tasks were evaluated: DEM vs. HC, where AD and FTD subjects were combined into a single DEM, and AD vs. FTD, where only the two dementia subgroups were included. The DEM vs. HC analysis was used to evaluate the ability of the EEG features to detect a broader dementia-related pattern, whereas the AD vs. FTD analysis was used to evaluate differential classification between dementia subtypes.

#### 2.6.2. Nested Cross-Validation and Feature Selection

To obtain an unbiased estimate of classification performance, nested cross-validation was used. The outer loop consisted of 5-fold cross-validation and was used exclusively to estimate the final model performance. Within each outer training fold, an inner 4-fold cross-validation loop was used for model selection. All procedures related to classification, including feature standardization, feature ranking, feature selection, and hyperparameter tuning, were performed exclusively within the training data of the corresponding cross-validation fold. This procedure was used to prevent information leakage from the test folds into the model training process.

Before model training, features were standardized using z-score normalization. For each training fold, the mean and standard deviation were calculated only from the training data. The same parameters were then applied to standardize the corresponding validation or test data. Feature selection was also performed within each training fold. In this context, feature reduction did not involve minimizing the numerical values of the features; instead, it involved reducing the dimensionality of the feature matrix by retaining only the top-ranked features. For three-class classification, features were ranked using the Kruskal–Wallis test calculated only on the training data. For binary classification, features were ranked using the Wilcoxon rank-sum test calculated only on the training data. Features were then sorted according to their training-fold statistical ranking, and only the top-ranked k features were retained for model training. The number of retained features, k, was treated as a model-selection parameter and was selected within the inner cross-validation loop. In the main three-class classification analyses, the tested numbers of selected features were (k = 5, 10, 20, 40, 80, 120, 160), and (240), with values exceeding the number of available features omitted. For the final long permutation testing, a reduced grid of (k = 5, 10, 20, 40), and (80) was used to reduce computational time.

#### 2.6.3. Classification Models and Performance Metrics

For the three-class task, a linear support vector machine classifier was implemented using an error-correcting output codes (ECOC) framework with one-vs-one coding [3]. For binary tasks, a direct linear support vector machine (SVM) was used [2]. The parameter (C), corresponding to the SVM box constraint, was selected in the inner cross-validation loop. In the main classification analyses, the tested values were (C = 0.01, 0.1, 1, and 10). Furthermore, for the final long permutation tests, a reduced grid of (C = 0.1) and (1) was used. Class prior probabilities were set to uniform in order to reduce the influence of class imbalance. No oversampling, undersampling, or synthetic sample generation was applied, because such procedures could introduce additional bias in a relatively small EEG dataset. Instead, the influence of unequal group sizes was mitigated by using uniform class priors during model training and by reporting class-balanced evaluation metrics.

Classification performance was quantified using balanced accuracy, macro-averaged F1-score, class-specific sensitivity, and confusion matrices. Balanced accuracy was selected as the primary metric because the dataset contained unequal numbers of AD, FTD, and HC subjects. For each class, sensitivity was calculated as the proportion of correctly classified subjects within that class. Macro-F1 was calculated as the unweighted average of F1-score values across all classes, providing a performance measure that gives equal importance to each diagnostic group. To further assess model stability, balanced accuracy and macro-F1 were also calculated separately for each outer cross-validation fold for the three main models selected for permutation testing. These fold-wise values were summarized as mean ± standard deviation across the five outer folds. In addition, 95% bootstrap confidence intervals were calculated from the subject-level out-of-fold predictions using 1000 bootstrap resamples. Because pooled out-of-fold metrics and fold-wise averaged metrics are calculated differently, the fold-wise mean values are intended to describe variability across folds and are not necessarily identical to the pooled performance values reported in the main classification table.

#### 2.6.4. Permutation Testing

Permutation testing was performed to evaluate whether the selected classification models performed above chance level. Three models were tested: the spectral-only three-class model, the combined GC + SPEC DEM vs. HC model, and the combined GC + spectral AD vs. FTD model. For each model, class labels were randomly permuted 1000 times, and the complete nested cross-validation procedure, including feature selection and hyperparameter tuning, was repeated for each permutation. The permutation *p*-value was calculated as

(4) $$p=\frac{1+\mathrm{count}\left(BA_{\mathrm{perm}}\geq BA_{\mathrm{obs}}\right)}{N_{\mathrm{perm}}+1}.$$

where (${\mathrm{BA}}_{obs}$) is the observed balanced accuracy obtained using the true diagnostic labels, (${\mathrm{BA}}_{perm}$) is the balanced accuracy obtained after randomly permuting the diagnostic labels, and ($N_{perm}=1000$) is the number of permutations. The term ($count(BA_{perm}\geq BA_{obs})$) denotes the number of permutations for which the permuted-label balanced accuracy was equal to or greater than the observed balanced accuracy. Conceptually, each permutation breaks the relationship between the EEG-derived feature matrix and the diagnostic labels while preserving the same feature values, class sizes, feature-selection procedure, hyperparameter tuning, and nested cross-validation structure. Therefore, Equation (4) estimates the empirical probability of obtaining a classification performance at least as high as the observed performance under the null hypothesis that the EEG features are not systematically related to the diagnostic labels. A small *p*-value indicates that the observed balanced accuracy is unlikely to be explained by random label assignment alone.

### 2.7. Exploratory PBM Case-Study Analysis

An exploratory single-case PBM analysis was performed as an external longitudinal extension of the main AD/FTD/HC EEG analysis. The PBM dataset consisted of repeated EEG recordings from a single case-study subject acquired during the photobiomodulation intervention protocol used in the previous study [6]. The previous PBM study focused on within-subject EEG changes associated with the PBM intervention. In contrast, the present manuscript uses the PBM recordings only as an exploratory external case-study projection into the spectral, GC, and combined GC + SPEC feature spaces derived from the AD/FTD/HC reference dataset. Because the PBM subject was not part of the AD/FTD/HC reference dataset and was not clinically labeled as AD or FTD, this analysis was used only to examine longitudinal EEG changes and relative similarity to the reference feature space, not for diagnostic validation.

For the present analysis, twelve PBM EEG recordings were included, corresponding to two assessment sessions and three stimulation-related phases for each recording condition. Recordings were analyzed separately for eyes-open and eyes-closed conditions and for before-, during-, and after-stimulation phases. In the original PBM protocol, EEG was recorded before stimulation for 10 min, consisting of 5 min with eyes open and 5 min with eyes closed. EEG was then recorded during PBM and again after PBM treatment for 10 min, using the same eyes-open and eyes-closed structure. Longitudinal changes were evaluated by comparing the first and last assessments for four primary conditions: eyes-open before stimulation, eyes-closed before stimulation, eyes-open after stimulation, and eyes-closed after stimulation.

The PBM intervention followed the protocol described in the original case-study report [6]. Stimulation was delivered using 810 nm LEDs pulsed at 10 Hz. During the first week, three stimulation sessions were performed at power level 2, while the remaining intervention period consisted of five sessions per week at power level 3. The intervention period lasted 35 days. These protocol details were used only to contextualize the longitudinal EEG comparison; the present analysis focused on EEG-derived spectral and directed connectivity changes between the first and last PBM assessments.

PBM EEG recordings were preprocessed using the same general preprocessing framework as the main AD/FTD/HC EEG dataset. The PBM recordings contained 16 EEG channels, whereas the main AD/FTD/HC dataset contained 19 channels. For the within-PBM longitudinal analysis, all 16 PBM channels were used, including channel-level, regional, and global features. For projection into the AD/FTD/HC reference feature space, only global and regional features were used, while electrode-level features were excluded to avoid direct comparison of non-identical montages. Spectral features were extracted from the preprocessed PBM signals using the same frequency bands as in the main analysis and included absolute power, relative power, and the TAR, DAR, and BARs.

GC-derived directed connectivity features were extracted from the preprocessed PBM recordings using the same frequency bands and matrix interpretation as in the main analysis. For each recording and frequency band, GC matrices were summarized using global connectivity measures, channel-level incoming and outgoing directed-influence measures, net-flow measures, and regional source-to-target averages. Longitudinal PBM changes were quantified as absolute and percent changes between the first and last assessments for the four primary conditions. To avoid unstable percent-change estimates caused by near-zero baseline values, robust GC summaries were additionally computed after excluding features with very small baseline values.

To explore the relationship between the PBM EEG recordings and the reference AD/FTD/HC feature space, centroid-based projection analyses were performed using spectral features, GC features, and combined GC + SPEC features, following the general principle of nearest-centroid approaches [17]. Reference centroids were calculated from the AD, FTD, and HC groups after standardization based only on the AD/FTD/HC dataset. Each PBM recording was then projected into this standardized feature space, and its distance to each group centroid was converted into a relative similarity percentage. These values were interpreted as exploratory similarity measures rather than diagnostic probabilities.

All PBM analyses were descriptive and exploratory. No inferential statistical testing was performed for the PBM dataset because it consisted of a single case-study subject. The PBM results were therefore interpreted as longitudinal within-subject EEG changes and as exploratory similarity to the AD/FTD/HC reference feature space. They were not used to assign a diagnostic label or to validate the AD/FTD/HC classifier.

## 3. Results

The results are presented in four parts. First, exploratory statistical results for spectral EEG features are presented. Then, group differences in GC-based directed connectivity features are presented. Furthermore, the results of the classification analyses performed using nested cross-validation are summarized for the three-class and binary classification tasks. Finally, permutation testing results are presented for the selected classification models.

### 3.1. Three-Group Differences in Spectral Features

Exploratory three-group statistical analysis of spectral features showed significant differences among AD, FTD, and HC subjects. The most pronounced differences were observed for TAR features, particularly at frontal electrodes and at the global level. The lowest *p*-values were obtained for TAR at electrodes F4, Fp2, the frontal region, F3, Fz, and Fp1. All presented top-ranked spectral features remained significant after Benjamini–Hochberg FDR correction.

Table 1 shows the top-ranked spectral features obtained by Kruskal–Wallis analysis across the three diagnostic groups.

**Figure 2:**
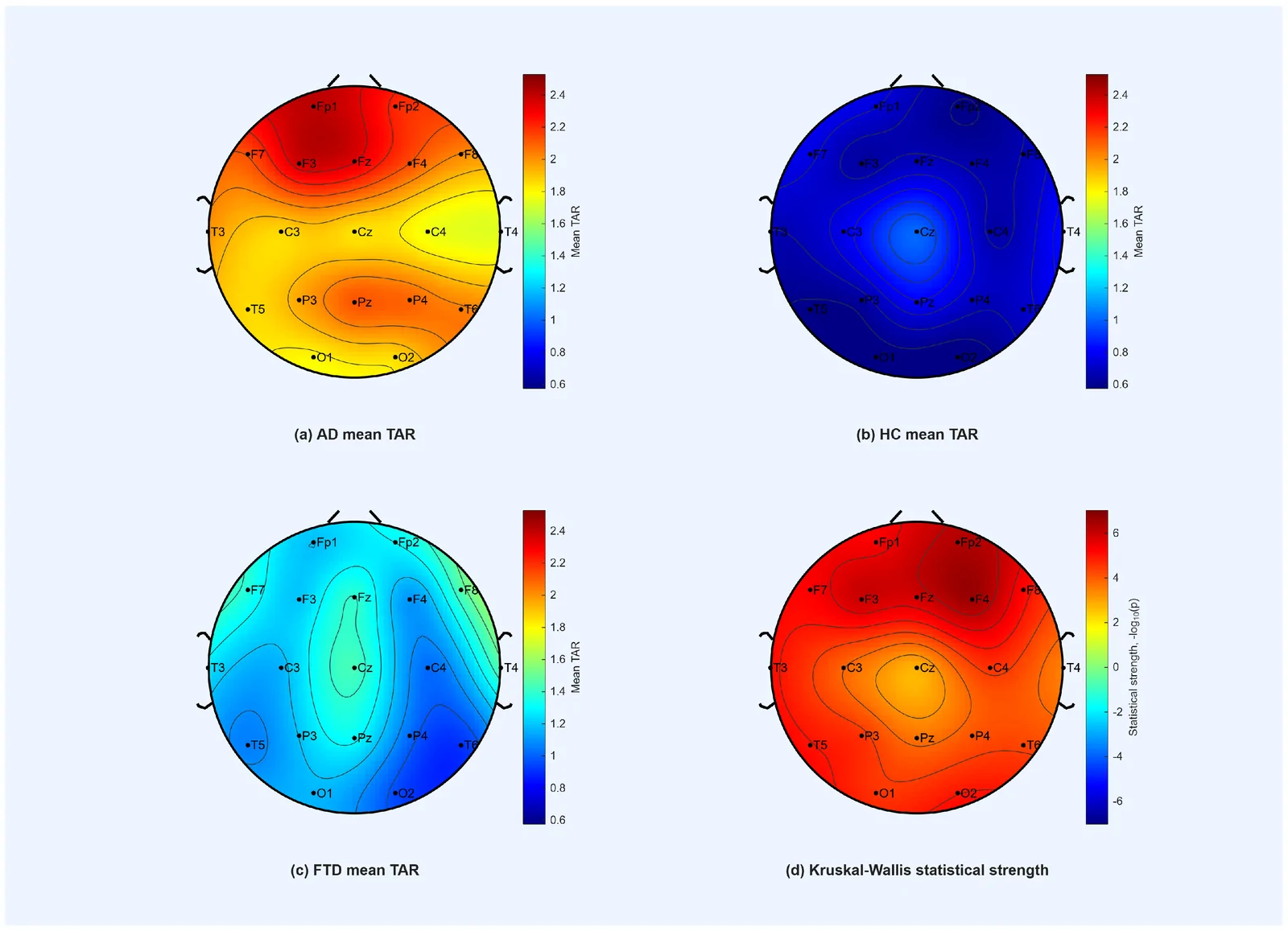
Topographic visualization of TAR-related spectral features across the three diagnostic groups. Panels show (**a**) mean TAR values for AD subjects, (**b**) mean TAR values for HC subjects, (**c**) mean TAR values for FTD subjects, and (**d**) the statistical strength of the three-group Kruskal–Wallis comparison expressed as $-\log_{10}(p)$, where larger values indicate smaller *p*-values and stronger group differences.

To complement the ranked statistical results in Table 1, the electrode-level TAR results are visualized topographically in Figure 2. The figure shows the group-level mean TAR distributions for AD, HC, and FTD subjects, together with the spatial distribution of the three-group Kruskal–Wallis statistical strength across electrodes.

### 3.2. Pairwise Spectral Feature Differences

Pairwise comparisons of spectral features were performed for AD vs. HC, HC vs. FTD, and AD vs. FTD. In the AD vs. HC comparison, the top-ranked features were TAR at electrodes F4, Fp2, the frontal region, Fz, F3, and the global level, with higher mean values observed in the AD group. In the HC vs. FTD comparison, the top-ranked features were TAR in the frontal region and at electrodes F7, Fp2, F3, F8, and the global level, with higher mean values observed in the FTD group. In the AD vs. FTD comparison, the top-ranked features were TAR at electrodes T6, F4, Fp1, O2, the frontal region, P4, T5, T3, F3, and Fp2, with higher mean values observed in the AD group. Table 2 shows the top-ranked pairwise differences in spectral features for each group comparison.

Table 2 further clarifies which diagnostic contrasts contributed to the spectral differences observed in the three-group analysis. Across all pairwise comparisons, the dominant features were TAR-based measures, indicating that the relative balance between theta and alpha activity was the most consistent spectral descriptor of group differences. In the AD vs. HC comparison, TAR values were higher in AD than in HC across frontal and global features, for example at F4, Fp2, frontal, Fz, F3, and global levels. This pattern is consistent with increased EEG slowing in AD. In the HC vs. FTD comparison, TAR values were also higher in FTD than in HC, particularly in frontal and frontotemporal electrodes such as F7, Fp2, F3, and F8, suggesting that TAR also captured dementia-related slowing in the FTD group. In the AD vs. FTD comparison, higher TAR values were observed in AD than in FTD for the listed features, including T6, F4, Fp1, O2, frontal, P4, T5, T3, F3, and Fp2. Therefore, Table 2 shows that TAR separated both dementia groups from HC, while also indicating that AD generally showed stronger TAR elevation than FTD in the pairwise dementia-subtype comparison. The contribution of Table 2 is therefore to demonstrate that the spectral findings were not limited to the overall three-group test, but were supported by consistent pairwise patterns across diagnostic contrasts.

### 3.3. Dementia vs. Healthy Control Spectral Differences

An additional binary comparison was performed between the combined DEM, consisting of AD and FTD subjects, and HC subjects. The top-ranked spectral features in this comparison were TAR at Fp2, the frontal region, F4, F3, Fz, F7, F8, and the global level. For all listed features, higher mean values were observed in the DEM group compared with the HC group. These features remained significant after FDR correction. Table 3 shows the top-ranked spectral differences for the DEM vs. HC comparison.

Although DAR and BAR were also included in the extracted spectral feature set, the top-ranked spectral results were dominated by TAR features; therefore, only TAR-based features appear among the leading results reported in Table 1, Table 2 and Table 3.

### 3.4. Three-Group Differences in GC-Derived Directed Connectivity Features

Exploratory three-group statistical analysis of GC-derived directed connectivity features was performed across AD, FTD, and HC subjects. The lowest *p*-values were obtained predominantly for alpha-band directed connectivity features, particularly for regional information flow toward temporal and frontal regions. The top-ranked features included alpha-band Frontal-to-temporal, Occipital-to-Temporal, Parietal-to-Temporal, and Parietal-to-Frontal connectivity, as well as low-beta and high-beta connectivity features. However, none of the listed GC-derived features reached the FDR-corrected significance threshold of (q<0.05). Table 4 shows the top-ranked GC-derived directed connectivity features obtained from the Kruskal–Wallis analysis across the three diagnostic groups.

Table 4 provides an exploratory overview of the GC-derived features that showed the strongest three-group differences among AD, HC, and FTD subjects. The leading features were dominated by alpha-band regional directed connectivity descriptors. In particular, the three lowest *p*-values were obtained for Alpha Frontal-to-Temporal, Alpha Occipital-to-Temporal, and Alpha Parietal-to-Temporal connectivity, indicating that directed interactions toward temporal regions were prominent among the top-ranked GC descriptors. Additional alpha-band features involved Parietal-to-Frontal, Parietal-to-Central, Frontal-to-Frontal, and Temporal-to-Temporal connectivity, suggesting that the strongest GC-related group trends were mainly distributed across regional alpha-band networks rather than being restricted to a single electrode pair or frequency band. Low-beta features also appeared among the top-ranked results, including Low-Beta Global Maximum and Low-Beta Occipital-to-Temporal connectivity, but the overall pattern was more strongly represented by alpha-band directed connectivity measures. Importantly, although these features showed low uncorrected *p*-values, none reached the FDR-corrected significance threshold of q < 0.05. Therefore, Table 4 should be interpreted as identifying exploratory GC-derived three-group trends that motivated further pairwise analysis, rather than as confirming statistically significant three-group GC biomarkers.

**Figure 3:**
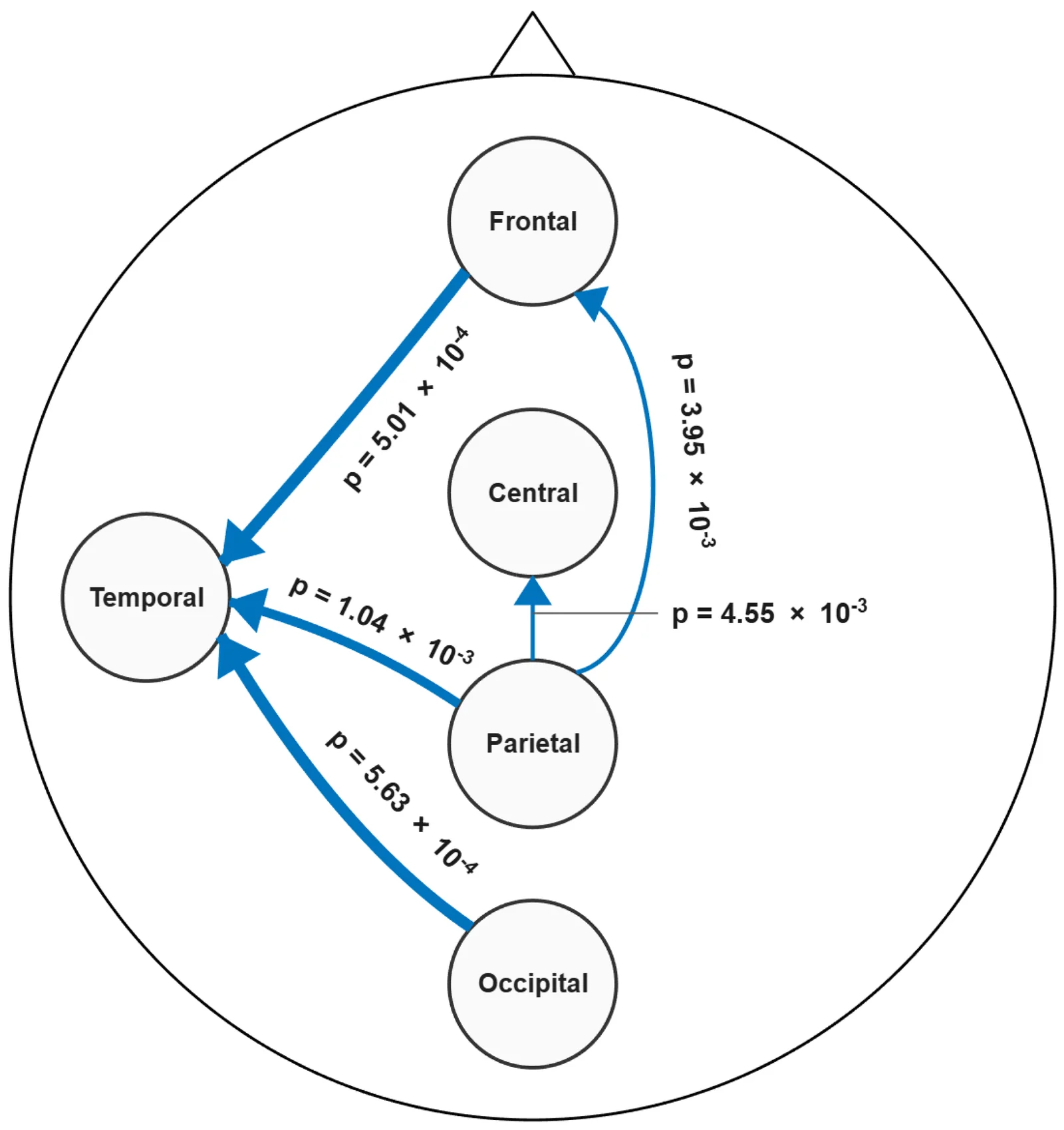
Node-edge diagram of the top-ranked regional alpha-band GC-derived directed connectivity descriptors identified in the three-group and pairwise analyses. Nodes represent scalp-electrode groupings based on the 10–20 montage, and arrows indicate source-to-target direction. Arrow width reflects the statistical ranking of the regional descriptors based on $-\log_{10}(p)$, where smaller uncorrected *p*-values are shown with thicker arrows, and should not be interpreted as the absolute magnitude of the GC value. Because the analysis was performed using bivariate sensor-level GC, the diagram should be interpreted as a descriptive sensor-level summary rather than as evidence of direct anatomical causal pathways.

To provide a visual summary of these directed connectivity patterns, the main regional GC-derived source-to-target descriptors are shown as a node-edge diagram in Figure 3. The diagram summarizes the strongest regional alpha-band directed connectivity descriptors from the three-group analysis and the pairwise comparisons. Only regional source-to-target descriptors are shown, because global descriptors and within-region descriptors cannot be represented as single directed inter-regional edges in the same way. Arrow width reflects the statistical ranking of the regional descriptors based on $-\log_{10}(p)$, where smaller uncorrected *p*-values are shown with thicker arrows. Therefore, the arrow width should not be interpreted as the absolute magnitude of the GC value. Because the GC analysis was performed using bivariate sensor-level models, the diagram should be interpreted as a descriptive scalp-level summary rather than as evidence of direct anatomical causal pathways.

### 3.5. Pairwise GC-Derived Directed Connectivity Differences

Pairwise comparisons of GC-derived directed connectivity features were performed for AD vs. HC, HC vs. FTD, and AD vs. FTD. In the AD vs. HC comparison, the top-ranked features included alpha-band Occipital-to-Temporal, Frontal-to-Temporal, Parietal-to-Temporal, and Frontal-to-Frontal connectivity, as well as low-beta global maximum connectivity. For these features, higher mean values were observed in the AD group than in the HC group, and the listed features remained significant after FDR correction.

In the HC vs. FTD comparison, the top-ranked features included alpha-band Parietal-to-Central, Parietal-to-Frontal, Frontal-to-Temporal, and Frontal-to-Central connectivity. For these features, higher mean values were observed in the FTD group than in the HC group, and the listed features remained significant after FDR correction.

In the AD vs. FTD comparison, the top-ranked features included alpha-band Occipital-to-Temporal and Parietal-to-Temporal connectivity, high-beta Parietal-to-Temporal connectivity, and alpha-band Temporal-to-Occipital connectivity. Higher mean values were observed in the AD group for the listed features. The first two features remained significant after FDR correction at (q<0.05), while the remaining listed features did not reach the FDR-corrected significance threshold. Table 5 summarizes the top-ranked pairwise GC-derived directed connectivity differences for each group comparison.

### 3.6. Classification Results

Classification performance was evaluated using nested cross-validation for the three-class classification task and for the two binary classification tasks. In the three-class classification of AD, HC, and FTD subjects, the spectral-only feature set achieved the highest balanced accuracy and macro-F1 score among the three evaluated feature sets. The GC-only feature set achieved lower performance, while the combined GC + spectral feature set did not improve the three-class classification performance compared with spectral features alone.

For the binary DEM vs. HC task, the combined GC + spectral feature set achieved the highest balanced accuracy, followed by the spectral-only feature set. In this task, both spectral-only and combined GC + SPEC produced higher balanced accuracy than the GC-only feature set. For the AD vs. FTD task, the combined GC + SPEC feature set achieved the highest balanced accuracy and macro-F1 score among the evaluated feature sets. Table 6 summarizes the classification results for all classification tasks and feature sets.

**Figure 4:**
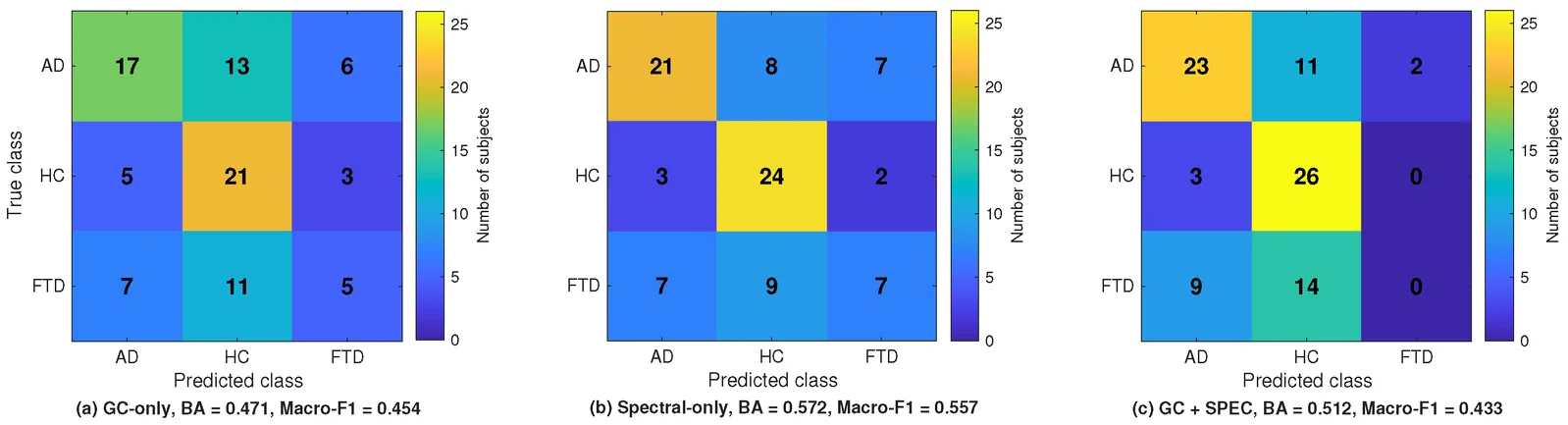
Confusion matrices for three-class AD/HC/FTD classification.

To further clarify the class-wise error structure of the three-class AD/HC/FTD classification models, confusion matrices are shown in Figure 4. These matrices complement the balanced accuracy, macro-F1, and sensitivity values reported in Table 6 by showing the direction of misclassification errors. In the combined GC + SPEC model, FTD sensitivity was 0.000, corresponding to 0 out of 23 correctly classified FTD subjects. The confusion matrix shows that 9 FTD subjects were misclassified as AD and 14 FTD subjects were misclassified as HC.

### 3.7. Permutation Testing Results

Permutation testing with 1000 label permutations was performed for three selected classification models: the spectral-only three-class model, the combined GC + SPEC DEM vs. HC model, and the combined GC + SPEC AD vs. FTD model. For each permutation, the complete nested cross-validation procedure was repeated, including feature selection and hyperparameter tuning.

The spectral-only three-class model achieved an observed balanced accuracy of 0.585 and an observed macro-F1 score of 0.575. The mean balanced accuracy obtained across permuted-label models was 0.334, with a standard deviation of 0.058. The permutation *p*-value for balanced accuracy was (*p* = 0.000999), corresponding to (*p* < 0.001).

For the DEM vs. HC task, the combined GC + SPEC model achieved an observed balanced accuracy of 0.710 and an observed macro-F1 score of 0.665. The mean balanced accuracy across permuted-label models was 0.500, with a standard deviation of 0.065. The permutation *p*-value for balanced accuracy was (*p* = 0.000999), corresponding to (*p* < 0.001).

For the AD vs. FTD task, the combined GC + SPEC model achieved an observed balanced accuracy of 0.548 and an observed macro-F1 score of 0.525 in the reduced-grid permutation-testing run. The mean balanced accuracy across permuted-label models was 0.501, with a standard deviation of 0.075. The permutation *p*-value for balanced accuracy was *p* = 0.270. This observed value differs from the main nested cross-validation result reported in Table 6 (balanced accuracy = 0.584, macro-F1 = 0.559) because the final long permutation analysis used a reduced hyperparameter grid for computational feasibility. Table 7 summarizes the permutation testing results.

Because the dataset contained 88 participants and the outer cross-validation loop consisted of five folds, the stability of the three main classification models was further evaluated using fold-wise performance estimates and bootstrap confidence intervals. Table 8 summarizes the balanced accuracy and macro-F1 values obtained in each outer fold, the mean ± SD across outer folds, and 95% bootstrap confidence intervals calculated from the subject-level out-of-fold predictions. These fold-wise summaries complement the pooled out-of-fold metrics reported in Table 6 and are intended to describe fold-to-fold variability.

Because the long permutation analysis required repeating the complete nested cross-validation procedure 1000 times, it was performed using the reduced feature-number and SVM box-constraint grids described above. Therefore, the observed performance values reported in Table 7 correspond to this reduced-grid permutation-testing run and are not necessarily identical to the full-grid main nested-CV results reported in Table 6.

The fold-wise results indicate moderate variability across outer folds. The DEM vs. HC GC + SPEC model showed the most consistent performance among the evaluated models, with the lowest fold-wise standard deviation for both balanced accuracy and macro-F1. In contrast, the AD vs. FTD GC + SPEC model showed greater uncertainty, reflected by a wider bootstrap confidence interval for balanced accuracy and macro-F1. This supports the interpretation that DEM vs. HC classification was more stable than AD vs. FTD differentiation in the present dataset.

**Figure 5:**
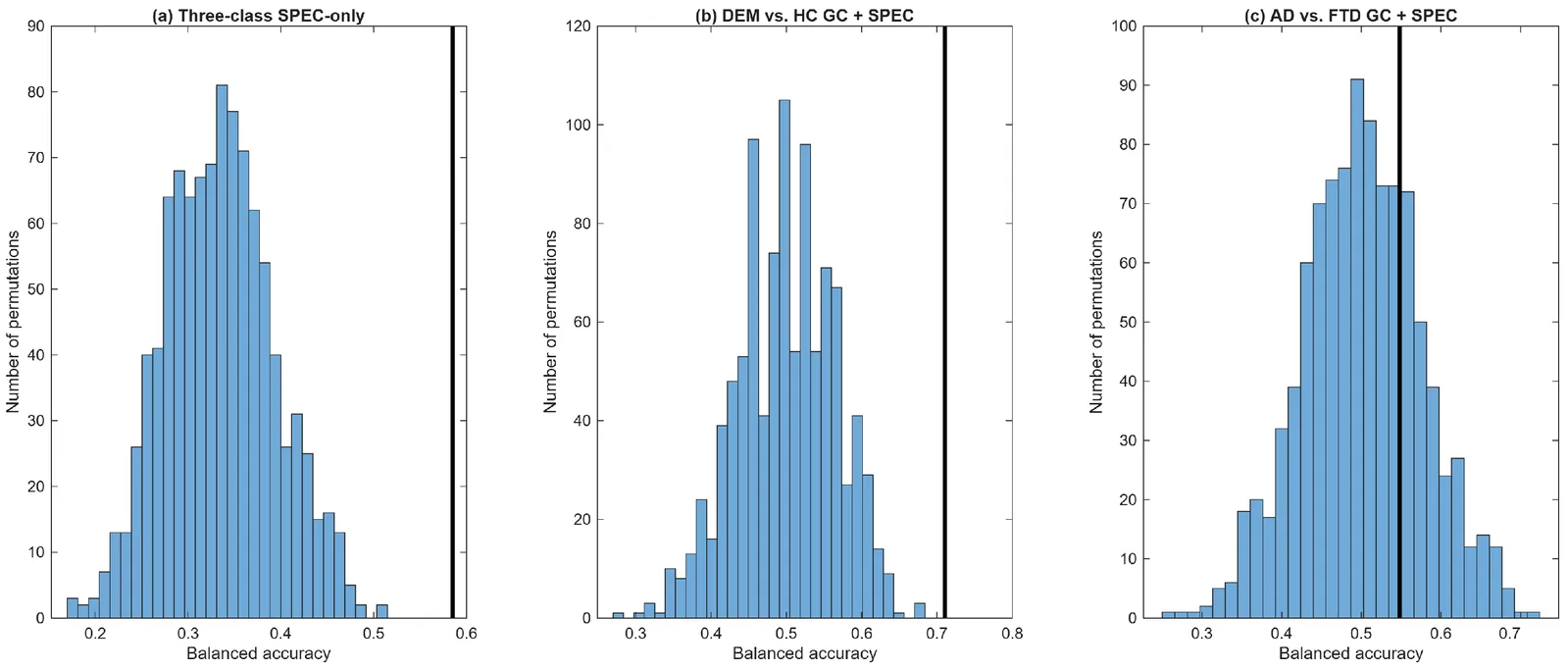
Permutation-test distributions for selected classification models. Panels show (**a**) the three-class spectral-only model, (**b**) the DEM vs. HC GC + SPEC model, and (**c**) the AD vs. FTD GC + SPEC model. Histograms represent balanced accuracy values obtained after label permutation, while the vertical black line indicates the observed balanced accuracy. The first two models showed observed performance above the permuted-label distribution, whereas the AD vs. FTD model overlapped with the permutation distribution.

The permutation-test distributions are visualized in Figure 5. For the three-class spectral-only model and the DEM vs. HC GC + SPEC model, the observed balanced accuracy values were located above the corresponding permuted-label distributions, indicating above-chance classification performance. In contrast, the observed balanced accuracy for the AD vs. FTD GC + SPEC model showed substantial overlap with the permutation distribution, supporting the non-significant permutation-test result reported in Table 7.

### 3.8. Exploratory PBM Results

The PBM case-study analysis included four first-to-last comparisons: eyes-open before stimulation, eyes-closed before stimulation, eyes-open after stimulation, and eyes-closed after stimulation. The results are summarized in Table 9.

Global alpha power increased from the first to the last PBM assessment in all four comparisons: by +5.04% when comparing eyes-open before-stimulation recordings from the first and last assessments, by +19.92% when comparing eyes-closed before-stimulation recordings from the first and last assessments, by +38.75% when comparing eyes-open after-stimulation recordings from the first and last assessments, and by +5.31% when comparing eyes-closed after-stimulation recordings from the first and last assessments. Global theta power also increased in all four comparisons: by +33.19%, +79.66%, +45.55%, and +61.97% in the same comparison order.

TAR increased from the first to the last PBM assessment in three comparisons: by +22.73% when comparing eyes-open before-stimulation recordings from the first and last assessments, by +34.88% when comparing eyes-closed before-stimulation recordings from the first and last assessments, and by +42.33% when comparing eyes-closed after-stimulation recordings from the first and last assessments. TAR decreased only when comparing eyes-open after-stimulation recordings from the first and last assessments (−2.58%). DAR decreased in all four comparisons: by −48.50% when comparing eyes-open before-stimulation recordings from the first and last assessments, by −17.80% when comparing eyes-closed before-stimulation recordings from the first and last assessments, by −42.31% when comparing eyes-open after-stimulation recordings from the first and last assessments, and by −23.72% when comparing eyes-closed after-stimulation recordings from the first and last assessments. BAR also decreased in all four comparisons: by −17.93%, −16.00%, −20.83%, and −14.09% in the same comparison order.

After excluding GC features with near-zero baseline values, the largest global or regional GC change in the eyes-open before-stimulation comparison was an increase in broadband global maximum connectivity of +161.97%. In the eyes-closed before-stimulation comparison, the largest retained change was an increase in broadband central-to-temporal connectivity of +260.05%. In the eyes-open after-stimulation comparison, the largest retained change was a decrease in broadband central-to-parietal connectivity of −91.17%. In the eyes-closed after-stimulation comparison, the largest change was an increase in broadband global maximum connectivity of +456.61%.

In the SPEC-only projection, the closest centroid was FTD, HC, FTD, and HC at the first assessment for the eyes-open before-stimulation, eyes-closed before-stimulation, eyes-open after-stimulation, and eyes-closed after-stimulation comparisons, respectively. At the last assessment, the closest centroid was AD, AD, FTD, and HC for the same four comparisons. In the GC-only and combined GC + SPEC projections, all four comparisons were closest to the HC centroid at both the first and last PBM assessments.

**Figure 6:**
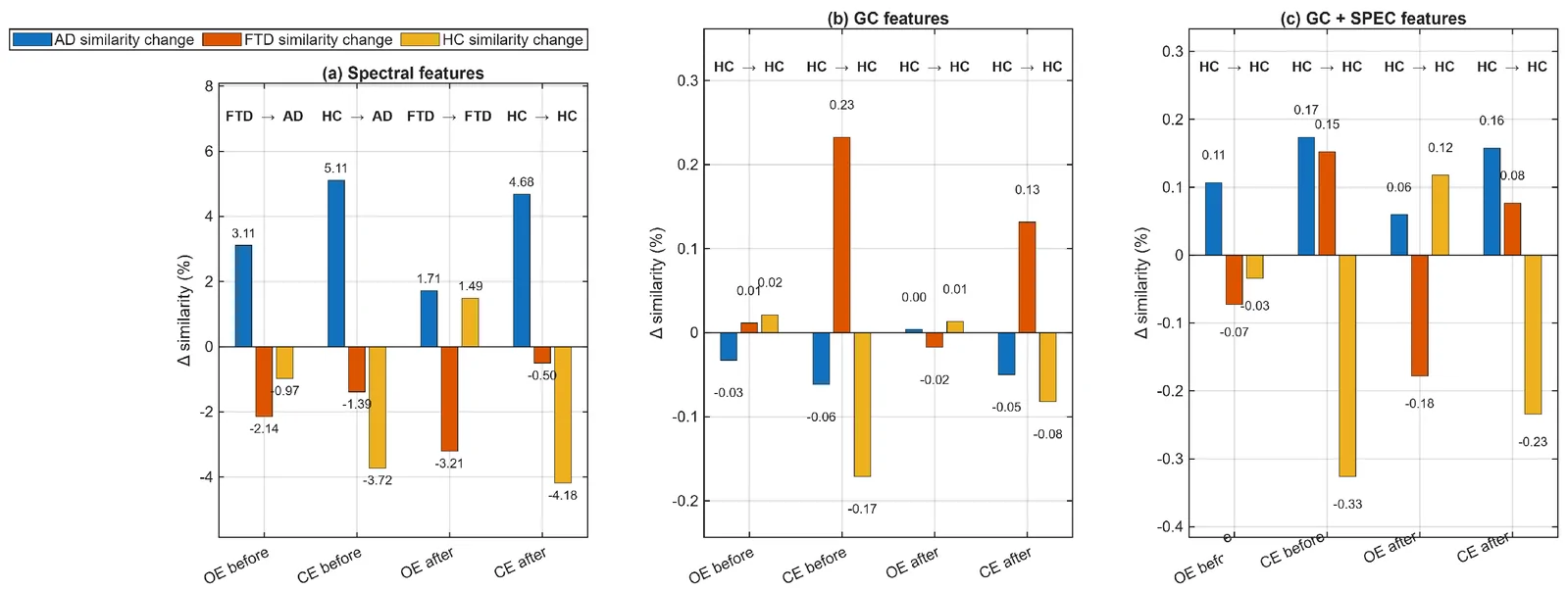
First-to-last changes in PBM centroid similarity across feature sets. Panels show (**a**) spectral features, (**b**) GC features, and (**c**) combined GC + SPEC features. Bars represent percentage changes in relative similarity to the AD, FTD, and HC reference centroids between the first and last PBM assessments for four comparison conditions: eyes-open before stimulation, eyes-closed before stimulation, eyes-open after stimulation, and eyes-closed after stimulation. The annotations above each group indicate the closest-centroid transition from the first to the last PBM assessment. These values are exploratory similarity measures and should not be interpreted as diagnostic probabilities.

To further support the interpretation of the PBM centroid analysis, first-to-last changes in relative similarity to the AD, FTD, and HC reference centroids are visualized in Figure 6. The figure shows the direction and magnitude of centroid-similarity changes for the spectral, GC, and combined GC + SPEC feature sets across the four PBM comparison conditions. This visualization complements the closest-centroid labels reported in Table 9 by showing that spectral-feature similarity changed more strongly across assessments, whereas GC-only and GC + SPEC similarities showed smaller changes and remained closest to the HC centroid across all four comparisons. Because the PBM analysis included a single case-study subject, these projection results are descriptive and should not be interpreted as diagnostic classification or diagnostic probability.

**Figure 7:**
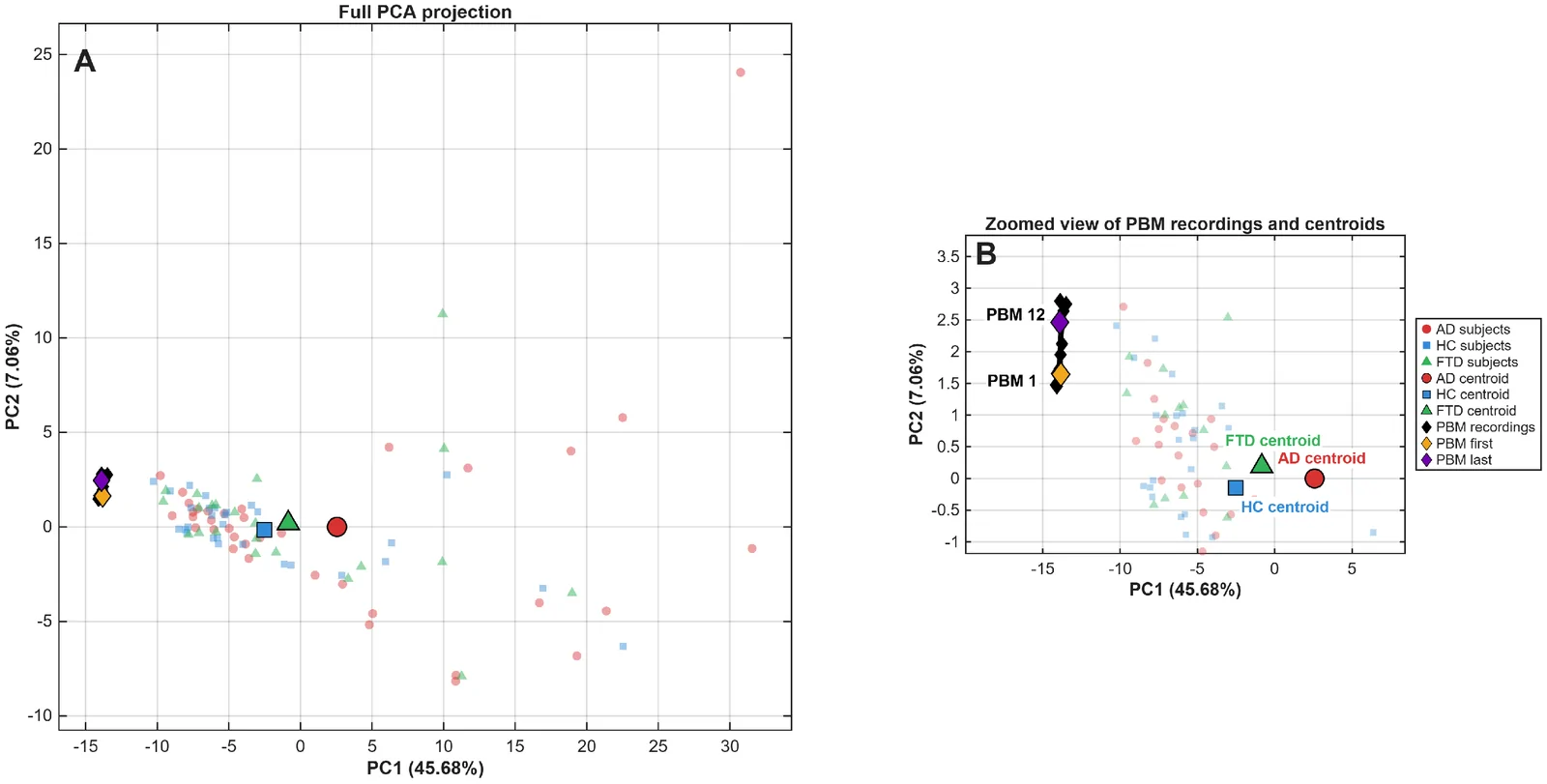
PCA projection of the exploratory PBM case-study assessments relative to the AD, FTD, and HC reference subjects and group centroids in the common GC + SPEC feature space. Panel (**A**) shows the full PCA projection, while panel (**B**) shows a zoomed view around the PBM recordings and group centroids. The PCA model was fitted on the reference AD/FTD/HC subjects only, and the PBM recordings were projected into this reference PCA space. Because the PBM dataset consisted of a single case-study subject and PCA shows only the first two principal components, this projection should be interpreted as a descriptive visualization rather than as diagnostic classification evidence.

To additionally support the centroid-based interpretation, a principal component analysis (PCA) projection of the reference AD/FTD/HC subjects and the PBM assessments is shown in Figure 7. The PCA model was fitted using only the reference AD/FTD/HC subjects, while the PBM recordings were subsequently projected into the same reference feature space using the common GC + SPEC features. This visualization provides a two-dimensional descriptive view of the feature-space relationships underlying the centroid-based similarity analysis.

In this projection, the PBM recordings are located in the same general region of the reference feature space as the HC centroid in the first two principal components. However, this visual pattern should be interpreted cautiously because the centroid analysis was performed in the full multidimensional feature space, whereas Figure 7 shows only a two-dimensional PCA projection.

## 4. Discussion

The present study evaluated spectral EEG features and GC-derived directed connectivity features for the characterization and classification of AD, FTD, and HC subjects. The main findings can be summarized in four points. First, spectral differences among the groups were dominated by TAR features, as shown in Table 1, Table 2 and Table 3. Second, GC-derived directed connectivity features showed group-related differences mainly in alpha-band regional connectivity, but the three-group GC analysis did not reach the FDR-corrected significance threshold, as shown in Table 4. The node-edge Figure 3 provides a visual summary of these regional source-to-target GC patterns, while preserving the interpretation of the findings as exploratory sensor-level directed connectivity descriptors. Third, classification results showed that spectral features provided the highest performance for the three-class AD vs. HC vs. FTD task, whereas the combination of GC and spectral features provided the highest balanced accuracy for the binary DEM vs. HC and AD vs. FTD tasks, as shown in Table 6. Fourth, permutation testing confirmed above-chance performance for the spectral-only three-class model and the combined GC + SPEC DEM vs. HC model, while the combined GC + SPEC AD vs. FTD model did not reach statistical significance, as shown in Table 7.

The present results should be interpreted in the context of previous EEG studies on dementia-related spectral slowing and functional-connectivity alterations. Prior work has shown that AD and FTD are associated with changes in oscillatory activity and network organization, but the relative contribution of spectral ratios and directed connectivity features remains task-dependent. The main contribution of the present study is the direct comparison of these feature families within a common preprocessing, validation, and statistical-testing framework. From an application perspective, TAR-based spectral markers may be useful as simple and interpretable EEG descriptors for separating dementia subjects from healthy controls, whereas GC-derived features may provide additional network-level information about regional directional interactions. However, these markers should be considered supportive research features rather than standalone diagnostic biomarkers. Their limitations include the relatively small sample size, unequal class sizes, scalp-level analysis, sensitivity of bivariate GC to common-source and volume-conduction effects, and the limited statistical support for AD vs. FTD differential classification. In addition, spectral and connectivity estimates may depend on the EEG referencing scheme. Therefore, the present results should be interpreted within the context of the average-reference preprocessing framework used in this study. Residual ocular contamination is another potential limitation, especially for frontal electrodes, because no separate EOG channels were available, and ICA/ICLabel-based artifact removal cannot guarantee complete elimination of all eye-related activity.

The spectral results showed that TAR was the most prominent feature type across the statistical analyses. In the three-group comparison, the lowest *p*-values were obtained for TAR features at frontal electrodes and at the global level, including TAR F4, TAR Fp2, TAR Frontal, TAR F3, TAR Fz, TAR Fp1, TAR F7, TAR F8, TAR T3, and TAR Global (Table 1). All of these features remained significant after FDR correction, with FDR-corrected *p*-values ranging from ($2.933\cdot 10^{-5}$) to ($1.265\cdot 10^{-4}$) (Table 1). In the DEM vs. HC comparison, the same general pattern was observed: TAR Fp2, TAR Frontal, TAR F4, TAR F3, TAR Fz, TAR F7, TAR F8, and TAR Global were all significant after FDR correction, with FDR-corrected *p*-values ranging from ($3.646\cdot 10^{-5}$) to ($1.473\cdot 10^{-4}$) (Table 3). These results indicate that, within this dataset and preprocessing pipeline, TAR-based features were the most statistically consistent spectral markers separating DEM subjects from HC.

The dominance of TAR among the spectral findings is consistent with previous EEG studies reporting dementia-related slowing, particularly increased theta activity and reduced alpha activity in AD [2,4]. Prior work on the same or related AD/FTD EEG datasets has also identified spectral ratios and connectivity features as relevant discriminative markers for distinguishing DEM from HC [2,5,8,13]. The present findings extend this line of work by showing that TAR remained the strongest statistical marker in the current feature set, while GC-derived directed connectivity captured additional regional network-level alterations, particularly in alpha-band interactions.

The two investigated EEG feature families have complementary advantages and disadvantages. Spectral features are relatively simple to compute, computationally efficient, and directly interpretable in terms of oscillatory slowing or activation within standard EEG frequency bands. In the present study, this was reflected by the strong and statistically robust performance of TAR-based features, especially for separating dementia subjects from HC. However, spectral features mainly describe the magnitude of local, regional, or global oscillatory activity and do not directly quantify the direction of information flow between brain regions. GC-derived directed connectivity features provide this additional network-level perspective by estimating whether past activity in one EEG signal improves the prediction of another signal. This can reveal source-to-target interactions that are not captured by power-based spectral measures alone. Nevertheless, GC-derived features are more methodologically demanding. They depend on preprocessing quality, autoregressive model-order selection, signal stationarity, sample size, and the reliability of pairwise model estimation. In scalp-level EEG, bivariate GC may also be influenced by common-source activity, volume conduction, or indirect interactions. Therefore, spectral features may be more robust and interpretable, whereas GC-derived features may provide complementary directed connectivity information but require more cautious interpretation.

Pairwise spectral comparisons further showed that the direction of TAR differences was not identical across all diagnostic contrasts. In AD vs. HC, higher mean TAR values were observed in AD subjects for the top-ranked features; for example, TAR F4 was 2.1289 in AD and 0.6515 in HC, while TAR Global was 2.0296 in AD and 0.6963 in HC (Table 2). In HC vs. FTD, higher mean TAR values were observed in FTD subjects; for example, TAR Frontal was 1.2839 in FTD and 0.6760 in HC, while TAR Global was 1.2068 in FTD and 0.6963 in HC (Table 2). In AD vs. FTD, higher mean TAR values were observed in AD subjects for the listed features, including TAR T6, TAR F4, TAR Fp1, TAR O2, TAR Frontal, TAR P4, TAR T5, TAR T3, TAR F3, and TAR Fp2 (Table 2). For example, TAR Frontal was 2.2296 in AD and 1.2839 in FTD (Table 2). Thus, TAR separated both DEM from HC, but AD generally showed higher TAR values than FTD in the pairwise AD vs. FTD spectral comparison.

The GC-derived directed connectivity analysis provided complementary information to the spectral analysis, but with weaker three-group statistical evidence after correction for multiple comparisons. In the three-group GC analysis, the lowest *p*-values were obtained mainly for regional alpha-band features, including Alpha Frontal-to-Temporal, Alpha Occipital-to-Temporal, Alpha Parietal-to-Temporal, and Alpha Parietal-to-Frontal connectivity (Table 4). However, none of the listed three-group GC features reached (q<0.05), with the lowest FDR-corrected *p*-value equal to ($5.766\cdot 10^{-2}$) (Table 4). This result suggests that GC-derived features showed detectable uncorrected group differences, but that these differences were not sufficiently strong to survive FDR correction in the three-group exploratory analysis.

In contrast, pairwise GC-derived comparisons identified several FDR-significant directed connectivity differences. In AD vs. HC, alpha-band connectivity features were higher in AD than in HC, including Alpha Occipital-to-Temporal, Alpha Frontal-to-Temporal, Alpha Parietal-to-Temporal, and Alpha Frontal-to-Frontal connectivity (Table 5). For example, Alpha Occipital-to-Temporal connectivity was 1.1513 in AD and 0.4504 in HC, with an FDR-corrected *p*-value of ($1.777\cdot 10^{-2}$) (Table 5). In HC vs. FTD, higher values were observed in FTD than in HC for Alpha Parietal-to-Central, Alpha Parietal-to-Frontal, Alpha Frontal-to-Temporal, and Alpha Frontal-to-Central connectivity (Table 5). For example, Alpha Frontal-to-Central connectivity was 0.8421 in FTD and 0.3094 in HC, with an FDR-corrected *p*-value of ($3.378\cdot 10^{-2}$) (Table 5). In AD vs. FTD, Alpha Occipital-to-Temporal and High-Beta Parietal-to-Temporal connectivity remained significant after FDR correction, with higher mean values in AD (Table 5). In particular, AD is often linked to posterior and distributed network disruption, whereas FTD is more strongly associated with frontal and temporal systems [4,7,8]. These findings indicate that GC-derived features captured directed connectivity differences that were more evident in pairwise comparisons than in the overall three-group test.

The classification results were consistent with the statistical feature analyses in showing a stronger contribution of spectral features to the three-class classification task. In AD vs. HC vs. FTD classification, the spectral-only feature set achieved the highest balanced accuracy and macro-F1 score among the evaluated feature sets, with balanced accuracy of 0.572 and macro-F1 of 0.557 (Table 6). The GC-only model achieved lower performance, with balanced accuracy of 0.471 and macro-F1 of 0.454 (Table 6). The combined GC + SPEC model did not improve three-class performance, reaching balanced accuracy of 0.512 and macro-F1 of 0.433 (Table 6). In this combined model, FTD sensitivity was 0.000, corresponding to 0 out of 23 correctly classified FTD subjects in the three-class setting (Table 6). The corresponding confusion matrix showed that these FTD subjects were misclassified as AD in 9 cases and as HC in 14 cases (Figure 4). Therefore, for the direct three-class task, adding GC-derived features to the spectral features did not improve classification performance.

For the binary DEM vs. HC classification task, the best balanced accuracy was obtained using the combined GC + SPEC feature set. This model achieved balanced accuracy of 0.710 and macro-F1 of 0.665, compared with 0.702 and 0.663 for the spectral-only model and 0.589 and 0.583 for the GC-only model (Table 6). The improvement of the combined model over the spectral-only model was numerically small, but the combined feature set achieved the highest value among the tested feature sets (Table 6). For the AD vs. FTD task, the combined GC + SPEC feature set also achieved the highest balanced accuracy and macro-F1 score among the three evaluated feature sets, with balanced accuracy of 0.584 and macro-F1 of 0.559 (Table 6). However, this performance remained modest, and class sensitivities showed an imbalance between AD and FTD, with AD sensitivity of 0.472 and FTD sensitivity of 0.696 (Table 6).

The moderate classification performance observed in this study should also be interpreted in the context of recent EEG-based AD/FTD classification literature. Several previous studies have reported higher classification accuracies on the same public dataset, but many of them used epoch-based segmentation, different validation schemes, or less conservative evaluation strategies [2,5,8,13]. Recent work has emphasized that subject-level validation and careful separation of training and test data are essential in small EEG datasets, because epoch-level leakage can lead to overly optimistic performance estimates [8]. Therefore, the more modest performance observed here may reflect a stricter evaluation setting rather than a lack of neurophysiological information in the extracted features.

To make this comparison more explicit, Table 10 summarizes selected EEG-based AD/FTD/HC classification studies performed on the same public resting-state EEG dataset or on closely related AD/FTD EEG datasets. The table compares the data source, preprocessing or denoising strategy, feature families and feature-selection approach, classifier and validation framework, reported performance, and main methodological considerations relevant to the present GC-based analysis. The aim of this comparison is not to rank studies only by classification accuracy, but to clarify differences in feature families, validation strategies, and statistical safeguards that affect the interpretation of reported performance. The aim of this comparison is not to rank studies only by classification accuracy, but to clarify differences in feature families, validation strategies, and limitations that affect the interpretation of reported performance.

Table 10 shows that the impact of GC in the present study should be interpreted as complementary rather than uniformly performance-improving. GC-derived features provided additional network-level information and contributed to the highest numerical performance in the DEM vs. HC task when combined with spectral features, but they did not improve the three-class model and did not yield statistically significant AD vs. FTD classification under permutation testing. Compared with previous studies reporting higher classification accuracies, the present work emphasizes subject-level nested validation, permutation-based statistical testing, and cautious interpretation of scalp-level GC-derived directed connectivity features. Direct numerical comparison across studies should therefore be made cautiously, because the reported metrics, validation schemes, feature-selection procedures, and statistical testing strategies were not identical.

The additional outer-fold stability analysis further showed that classification performance was not uniform across folds. This variability is important because each outer fold contained a relatively small number of subjects, especially from the FTD group. The DEM vs. HC GC + SPEC model showed comparatively stable performance across folds, whereas the AD vs. FTD GC + SPEC model showed wider uncertainty. Therefore, the pooled balanced accuracy and macro-F1 values should be interpreted together with the fold-wise mean ± SD and bootstrap confidence intervals. This is particularly relevant for the AD vs. FTD task, where the numerical advantage of the combined GC + SPEC model was not supported by permutation testing.

Permutation testing provided an additional validation step for the selected classification models. The spectral-only three-class model achieved an observed balanced accuracy of 0.585, compared with a permuted-label mean balanced accuracy of (0.334±0.058), with (*p* < 0.001) (Table 7). The combined GC + SPEC DEM vs. HC model achieved an observed balanced accuracy of 0.710, compared with a permuted-label mean of (0.500±0.065), also with (*p* < 0.001) (Table 7). These results show that both selected models performed above chance level under the permutation-testing framework. In contrast, the combined GC + SPEC AD vs. FTD model achieved observed balanced accuracy of 0.548 in the reduced-grid permutation-testing run, compared with a permuted-label mean of 0.501±0.075, with *p* = 0.270 (Table 7). This value should be distinguished from the full-grid main nested-CV result for the same feature set, which achieved balanced accuracy of 0.584 and macro-F1 of 0.559 in Table 6. Therefore, although the combined GC + SPEC feature set gave the highest numerical performance for AD vs. FTD among the tested feature sets, its performance was not statistically significant in the permutation test.

Taken together, these findings indicate that TAR-based spectral features were the most robust markers in this analysis, especially for separating dementia subjects from healthy controls. GC-derived directed connectivity features contributed additional pairwise information, particularly in alpha-band regional connections, and improved the numerical performance of the binary DEM vs. HC and AD vs. FTD models when combined with spectral features. However, the improvement was not uniform across tasks. The combined GC + SPEC feature set did not improve the three-class classification and did not yield statistically significant AD vs. FTD classification under permutation testing. Therefore, the present results support the usefulness of spectral EEG markers, particularly TAR, and suggest that GC-derived directed connectivity may provide complementary information, but they do not support a conclusion that GC + spectral integration is sufficient for reliable AD vs. FTD discrimination in this dataset.

Several limitations should be considered. First, the dataset included 88 subjects, with unequal group sizes: 36 AD, 23 FTD, and 29 HC subjects. This group imbalance was mitigated by using uniform class priors, balanced accuracy, macro-F1, class-specific sensitivity, and confusion matrices, rather than by applying oversampling, undersampling, or synthetic sample generation. However, the imbalance was not fully eliminated and remains relevant when interpreting class-specific sensitivity, especially for the smaller FTD group and for the AD vs. FTD differential classification task. Second, all analyses were performed at the scalp-electrode level. Therefore, GC-derived directed connectivity should be interpreted as predictive directional interaction between EEG sensor signals, not as direct anatomical causation between cortical sources. Third, the statistical analyses were exploratory and involved multiple feature comparisons; therefore, FDR correction was applied, and only corrected findings should be considered statistically robust. Fourth, the final permutation tests were performed on selected classification models, rather than on all tested feature-set and task combinations. Finally, the AD vs. FTD result indicates that additional methodological development, larger datasets, or complementary feature types may be required for reliable differential classification between dementia subtypes.

However, these GC findings should be interpreted with caution because the present analysis used bivariate pairwise GC at the sensor level. In this framework, each source-target electrode pair was evaluated independently of the remaining electrodes. Therefore, common neural sources, volume conduction, or indirect interactions may contribute to apparent directed relationships, particularly between spatially close electrodes or neighboring regions. For this reason, the GC-derived features should be considered predictive directed connectivity markers useful for group characterization and classification, rather than direct evidence of anatomical causal pathways.

The exploratory PBM case-study analysis provided a longitudinal extension of the EEG markers examined in the main AD/FTD/HC dataset. This part of the study should also be distinguished from the previous PBM case-study report [6]. The previous study focused on within-subject spectral and connectivity changes associated with the PBM intervention itself. In contrast, the present manuscript uses the PBM recordings only as an exploratory external projection into the spectral, GC, and combined GC + SPEC feature spaces derived from the AD/FTD/HC reference dataset. While the main analysis focused on group-level differences and classification among AD, FTD, and HC participants, the PBM analysis examined within-subject EEG changes between the first and last PBM assessment. Therefore, the PBM findings should be interpreted as complementary longitudinal observations and descriptive similarity projections rather than as an independent validation of the diagnostic classification framework.

The most consistent spectral finding in the PBM analysis was an increase in global alpha power across all four first-to-last comparisons, ranging from +5.04% to +38.75% (Table 9). This is relevant because reduced alpha activity and increased slow-wave activity are commonly associated with cognitive impairment and dementia-related EEG slowing [6,14]. However, global theta power also increased in all comparisons, ranging from +33.19% to +79.66%, which led to increased TAR in three of the four conditions (Table 9). Therefore, the PBM spectral results do not indicate a uniform normalization of the theta-to-alpha profile. Instead, they suggest a mixed spectral pattern, combining increased alpha activity with a concurrent increase in theta power.

In contrast to TAR, both DAR and BAR decreased consistently across all four PBM comparisons (Table 9). DAR decreased by 17.80% to 48.50%, suggesting a reduction in delta activity relative to alpha activity, which may be interpreted as a potentially favorable spectral shift. The most favorable overall spectral pattern was observed in the eyes-open after-stimulation condition, where alpha increased by 38.75%, TAR decreased by 2.58%, DAR decreased by 42.31%, and BAR decreased by 20.83% (Table 9). This condition therefore showed the clearest combination of increased alpha activity and reduced ratio-based slowing markers.

The GC-derived features showed changes in directed connectivity descriptors between the first and last PBM assessments (Table 9). The largest retained relative GC changes involved broadband global or regional connectivity, ranging from a 91.17% decrease in broadband central-to-parietal connectivity to a 456.61% increase in broadband global maximum connectivity. However, these values should be interpreted cautiously because they represent relative percentage changes and may be strongly magnified when baseline GC values are small. Therefore, the largest relative increase should not be interpreted as proportional evidence of a large physiological effect. Instead, these results indicate exploratory changes in GC-derived directed connectivity descriptors between assessments. The magnitude and direction of an individual GC change cannot by themselves define whether the change is beneficial, because both increased and decreased directed influences may have different meanings depending on the affected network, frequency band, baseline value, and model-estimation characteristics.

The centroid-based projection analysis provided an additional exploratory perspective on the PBM EEG profiles. The SPEC-only projection was not stable across conditions, with the closest centroid changing between FTD, HC, and AD depending on the condition and assessment. In contrast, the GC-only and combined GC + SPEC projections remained closest to the HC centroid at both the first and last PBM assessments across all four comparisons (Table 9). This suggests that the global and regional directed connectivity profile, alone or combined with spectral features, was more consistently HC-like than the spectral profile alone. However, these centroid-based values represent exploratory similarity measures and should not be interpreted as diagnostic probabilities. The PCA projection, Figure 7, provides a visual check of this centroid-based analysis, but it remains only a two-dimensional descriptive representation of a multidimensional feature space and does not provide diagnostic validation.

Overall, the PBM findings should be viewed as exploratory observations of EEG changes in a single case. The consistent alpha increase, reduced DAR and BAR, GC-derived connectivity changes, and HC-like GC/GC + SPEC centroid projections suggest measurable longitudinal changes in the extracted EEG descriptors, rather than direct evidence of physiological normalization. However, the concurrent theta increase and the absence of consistent TAR reduction indicate that these changes do not represent a uniform normalization of the EEG profile. Because the PBM analysis included only one subject, no statistical inference or diagnostic conclusion can be drawn from these results.

## 5. Conclusions

This study examined EEG-derived spectral and GC-based directed connectivity features for the characterization and classification of AD, FTD, and HC subjects. The most robust statistical findings were obtained for spectral features, especially TAR, which dominated the three-group and pairwise spectral analyses. These results confirm the importance of EEG slowing markers, particularly frontal and global TAR, for distinguishing dementia-related EEG patterns from HC activity.

GC-derived directed connectivity features provided additional information about network-level alterations, particularly in alpha-band regional interactions. Although the strongest three-group GC effects did not reach FDR significance, pairwise analyses suggested that directed connectivity may capture complementary aspects of AD- and FTD-related network organization.

Classification performance was moderate rather than diagnostic-grade. The best three-class AD/FTD/HC result was obtained with spectral features, reaching a balanced accuracy of 0.572 and a macro-F1 score of 0.557. In binary classification, the combined GC + SPEC feature set performed best for DEM vs. HC, with a balanced accuracy of 0.710 and a macro-F1 score of 0.665. AD vs. FTD remained the most challenging task, with the best combined GC + SPEC model reaching a balanced accuracy of 0.584 and a macro-F1 score of 0.559. Permutation testing confirmed above-chance performance for the three-class spectral-only model and the DEM vs. HC GC + SPEC model, whereas AD vs. FTD did not reach statistical significance.

The exploratory PBM case-study analysis provided an additional longitudinal perspective on the extracted EEG markers. The PBM subject showed measurable EEG reorganization between the first and last assessment, including increased alpha power, consistent reductions in DAR and BAR, substantial GC-derived connectivity changes, and GC/GC + SPEC centroid projections closest to the HC reference space. However, because this analysis involved a single case and showed mixed TAR changes, it should be interpreted only as exploratory evidence of longitudinal EEG change, not as diagnostic validation.

Overall, the findings indicate that spectral EEG ratios and GC-derived directed connectivity provide complementary information for dementia-related EEG analysis. Spectral markers, particularly TAR, were the most discriminative in the present dataset, while directed connectivity contributed additional network-level information and improved selected binary classification settings. These findings support the potential use of EEG-derived spectral and directed connectivity descriptors as complementary research markers for dementia-related EEG characterization, while emphasizing that further validation on larger independent datasets is required before clinical application. However, because the present GC analysis was based on bivariate sensor-level models, future studies should extend this framework toward multivariate or conditional GC approaches, ideally combined with source-space reconstruction, to reduce the influence of common-source activity, volume conduction, and indirect interactions. Such extensions may provide more specific directed connectivity markers and improve the differential classification of AD and FTD. Future studies should also further investigate whether longitudinal EEG markers can support monitoring of disease progression or intervention-related neurophysiological change.

## Figures and Tables

**Table 1:** Top-ranked spectral features based on statistical significance.

Rank	Spectral Feature	*p*-Value	FDR-Corrected *p*-Value
1	TAR F4	$2.491\cdot 10^{-7}$	$2.933\cdot 10^{-5}$
2	TAR Fp2	$4.790\cdot 10^{-7}$	$2.933\cdot 10^{-5}$
3	TAR Frontal	$5.533\cdot 10^{-7}$	$2.933\cdot 10^{-5}$
4	TAR F3	$1.063\cdot 10^{-6}$	$4.224\cdot 10^{-5}$
5	TAR Fz	$1.354\cdot 10^{-6}$	$4.304\cdot 10^{-5}$
6	TAR Fp1	$4.670\cdot 10^{-6}$	$1.226\cdot 10^{-4}$
7	TAR F7	$5.398\cdot 10^{-6}$	$1.226\cdot 10^{-4}$
8	TAR F8	$6.312\cdot 10^{-6}$	$1.255\cdot 10^{-4}$
9	TAR T3	$7.467\cdot 10^{-6}$	$1.265\cdot 10^{-4}$
10	TAR Global	$7.953\cdot 10^{-6}$	$1.265\cdot 10^{-4}$

**Table 2:** Comparison of spectral features between diagnostic groups.

Comparison	Spectral Feature	Mean Group 1	Mean Group 2	*p*-Value	FDR-Corrected *p*-Value
AD vs. HC	TAR F4	2.1289	0.6515	$4.470\cdot 10^{-7}$	$2.601\cdot 10^{-5}$
AD vs. HC	TAR Fp2	2.2089	0.5924	$7.725\cdot 10^{-7}$	$2.601\cdot 10^{-5}$
AD vs. HC	TAR Frontal	2.2296	0.6760	$1.156\cdot 10^{-6}$	$2.601\cdot 10^{-5}$
AD vs. HC	TAR Fz	2.3366	0.6878	$1.156\cdot 10^{-6}$	$2.601\cdot 10^{-5}$
AD vs. HC	TAR F3	2.3662	0.6285	$1.720\cdot 10^{-6}$	$3.096\cdot 10^{-5}$
AD vs. HC	TAR Global	2.0296	0.6963	$7.006\cdot 10^{-6}$	$6.305\cdot 10^{-5}$
HC vs. FTD	TAR Frontal	0.6760	1.2839	$3.048\cdot 10^{-4}$	$1.016\cdot 10^{-3}$
HC vs. FTD	TAR F7	0.7578	1.3630	$3.048\cdot 10^{-4}$	$1.016\cdot 10^{-3}$
HC vs. FTD	TAR Fp2	0.5924	1.2900	$3.272\cdot 10^{-4}$	$1.052\cdot 10^{-3}$
HC vs. FTD	TAR F3	0.6285	1.1983	$4.973\cdot 10^{-4}$	$1.544\cdot 10^{-3}$
HC vs. FTD	TAR F8	0.6725	1.4535	$6.532\cdot 10^{-4}$	$1.960\cdot 10^{-3}$
HC vs. FTD	TAR Global	0.6963	1.2068	$2.672\cdot 10^{-3}$	$6.012\cdot 10^{-3}$
AD vs. FTD	TAR T6	2.0409	0.9071	$2.918\cdot 10^{-3}$	$6.405\cdot 10^{-3}$
AD vs. FTD	TAR F4	2.1289	1.1018	$3.228\cdot 10^{-3}$	$6.917\cdot 10^{-3}$
AD vs. FTD	TAR Fp1	2.4070	1.1856	$5.804\cdot 10^{-3}$	$1.161\cdot 10^{-2}$
AD vs. FTD	TAR O2	1.8596	0.9570	$9.235\cdot 10^{-3}$	$1.723\cdot 10^{-2}$
AD vs. FTD	TAR Frontal	2.2296	1.2839	$1.437\cdot 10^{-2}$	$2.536\cdot 10^{-2}$
AD vs. FTD	TAR P4	2.0883	1.0178	$1.566\cdot 10^{-2}$	$2.710\cdot 10^{-2}$
AD vs. FTD	TAR T5	1.8604	1.0580	$1.634\cdot 10^{-2}$	$2.769\cdot 10^{-2}$
AD vs. FTD	TAR T3	2.0069	1.2609	$1.778\cdot 10^{-2}$	$2.900\cdot 10^{-2}$
AD vs. FTD	TAR F3	2.3662	1.1983	$1.854\cdot 10^{-2}$	$2.900\cdot 10^{-2}$
AD vs. FTD	TAR Fp2	2.2089	1.2900	$1.933\cdot 10^{-2}$	$2.900\cdot 10^{-2}$

**Table 3:** Statistically significant spectral features after FDR correction.

Spectral Feature	*p*-Value	FDR-Corrected *p*-Value
TAR Fp2	$4.194\cdot 10^{-7}$	$3.646\cdot 10^{-5}$
TAR Frontal	$5.288\cdot 10^{-7}$	$3.646\cdot 10^{-5}$
TAR F4	$7.632\cdot 10^{-7}$	$3.646\cdot 10^{-5}$
TAR F3	$9.576\cdot 10^{-7}$	$3.646\cdot 10^{-5}$
TAR Fz	$1.147\cdot 10^{-6}$	$3.646\cdot 10^{-5}$
TAR F7	$2.894\cdot 10^{-6}$	$6.863\cdot 10^{-5}$
TAR F8	$3.022\cdot 10^{-6}$	$6.863\cdot 10^{-5}$
TAR Global	$7.675\cdot 10^{-6}$	$1.473\cdot 10^{-4}$

**Table 4:** Top-ranked GC-derived features based on statistical significance.

Rank	GC-Derived Feature	*p*-Value	FDR-Corrected *p*-Value
1	Alpha Frontal-to-Temporal	$5.010\cdot 10^{-4}$	$5.766\cdot 10^{-2}$
2	Alpha Occipital-to-Temporal	$5.630\cdot 10^{-4}$	$5.766\cdot 10^{-2}$
3	Alpha Parietal-to-Temporal	$1.045\cdot 10^{-3}$	$7.139\cdot 10^{-2}$
4	Alpha Parietal-to-Frontal	$3.947\cdot 10^{-3}$	$1.132\cdot 10^{-1}$
5	Low-Beta Global Maximum	$4.049\cdot 10^{-3}$	$1.132\cdot 10^{-1}$
6	Alpha Parietal-to-Central	$4.551\cdot 10^{-3}$	$1.132\cdot 10^{-1}$
7	Alpha Global Mean	$5.019\cdot 10^{-3}$	$1.132\cdot 10^{-1}$
8	Alpha Frontal-to-Frontal	$5.097\cdot 10^{-3}$	$1.132\cdot 10^{-1}$
9	Alpha Temporal-to-Temporal	$5.552\cdot 10^{-3}$	$1.132\cdot 10^{-1}$
10	Low-Beta Occipital-to-Temporal	$5.638\cdot 10^{-3}$	$1.132\cdot 10^{-1}$

**Table 5:** Comparison of GC-derived features between diagnostic groups.

Comparison	GC-Derived Feature	Group 1 Mean	Group 2 Mean	*p*-Value	FDR-Corrected *p*-Value
AD vs. HC	Alpha Occipital-to-Temporal	1.1513	0.4504	3.399 × $10^{-4}$	$1.777\times 10^{-2}$
AD vs. HC	Alpha Frontal-to-Temporal	1.1209	0.4365	$3.949\times 10^{-4}$	$1.777\times 10^{-2}$
AD vs. HC	Alpha Parietal-to-Temporal	1.1148	0.4144	$6.590\times 10^{-4}$	$1.977\times 10^{-2}$
AD vs. HC	Low-Beta Global Maximum	6.0806	4.3831	$1.574\times 10^{-3}$	$2.116\times 10^{-2}$
AD vs. HC	Alpha Frontal-to-Frontal	1.1930	0.3867	$1.647\times 10^{-3}$	$2.116\times 10^{-2}$
HC vs. FTD	Alpha Parietal-to-Central	0.2231	0.7814	$3.385\times 10^{-3}$	$2.168\times 10^{-2}$
HC vs. FTD	Alpha Parietal-to-Frontal	0.3548	0.7233	$6.036\times 10^{-3}$	$2.236\times 10^{-2}$
HC vs. FTD	Alpha Frontal-to-Temporal	0.4365	0.6119	$9.380\times 10^{-3}$	$2.888\times 10^{-2}$
HC vs. FTD	Alpha Frontal-to-Central	0.3094	0.8421	$1.239\times 10^{-2}$	$3.378\times 10^{-2}$
AD vs. FTD	Alpha Occipital-to-Temporal	1.1513	0.4856	$1.099\times 10^{-2}$	$3.090\times 10^{-2}$
AD vs. FTD	High-Beta Parietal-to-Temporal	1.3362	0.7712	$1.705\times 10^{-2}$	$4.384\times 10^{-2}$
AD vs. FTD	Alpha Temporal-to-Occipital	0.9148	0.5722	$3.046\times 10^{-2}$	$6.829\times 10^{-2}$
AD vs. FTD	Alpha Parietal-to-Temporal	1.1148	0.5245	$3.653\times 10^{-2}$	$7.646\times 10^{-2}$

**Table 6:** Classification performance for different feature sets and diagnostic tasks.

Classification Task	Feature Set	Balanced Accuracy	Macro-F1	Class Sensitivity
AD vs. HC vs. FTD	GC-only	0.471	0.454	AD: 0.472, HC: 0.724, FTD: 0.217
AD vs. HC vs. FTD	Spectral-only	0.572	0.557	AD: 0.583, HC: 0.828, FTD: 0.304
AD vs. HC vs. FTD	GC + SPEC	0.512	0.433	AD: 0.639, HC: 0.897, FTD: 0.000
DEM vs. HC	GC-only	0.589	0.583	DEM: 0.661, HC: 0.517
DEM vs. HC	Spectral-only	0.702	0.663	DEM: 0.610, HC: 0.793
DEM vs. HC	GC + SPEC	0.710	0.665	DEM: 0.593, HC: 0.828
AD vs. FTD	GC-only	0.440	0.435	AD: 0.444, FTD: 0.435
AD vs. FTD	Spectral-only	0.519	0.506	AD: 0.472, FTD: 0.565
AD vs. FTD	GC + SPEC	0.584	0.559	AD: 0.472, FTD: 0.696

**Table 7:** Permutation test results for selected classification models. Observed values in this table correspond to the reduced-grid permutation-testing run and therefore may differ from the full-grid main nested cross-validation values reported in Table 6.

Model	Observed Balanced Accuracy	Permuted Balanced Accuracy, Mean ± SD	*p*-Value for Balanced Accuracy	Observed Macro-F1	*p*-Value for Macro-F1
Three-class, spectral-only	0.585	0.334±0.058	<0.001	0.575	<0.001
DEM vs. HC, GC + SPEC	0.710	0.500±0.065	<0.001	0.665	0.003
AD vs. FTD, GC + SPEC	0.548	0.501±0.075	0.270	0.525	0.339

**Table 8:** Outer-fold variability and bootstrap confidence intervals for the three main classification models selected for permutation testing. Fold-wise values are reported across the five outer folds. Confidence intervals were calculated from subject-level out-of-fold predictions using 1000 bootstrap resamples.

Model	Task	Metric	Values Across Outer Folds	Mean ± SD	95% CI
Spectral-only	AD vs. HC vs. FTD	BA	0.635, 0.675, 0.498, 0.487, 0.563	0.572 ± 0.083	[0.474, 0.666]
Spectral-only	AD vs. HC vs. FTD	Macro-F1	0.634, 0.659, 0.493, 0.458, 0.785	0.605 ± 0.132	[0.445, 0.653]
GC + SPEC	DEM vs. HC	BA	0.735, 0.667, 0.625, 0.792, 0.750	0.714 ± 0.067	[0.616, 0.800]
GC + SPEC	DEM vs. HC	Macro-F1	0.702, 0.610, 0.600, 0.766, 0.646	0.665 ± 0.069	[0.561, 0.763]
GC + SPEC	AD vs. FTD	BA	0.661, 0.500, 0.586, 0.514, 0.686	0.589 ± 0.084	[0.444, 0.707]
GC + SPEC	AD vs. FTD	Macro-F1	0.633, 0.413, 0.580, 0.500, 0.667	0.559 ± 0.103	[0.424, 0.676]

**Table 9:** Summary of PBM-related changes in spectral ratios, GC-derived features, and classifier centroid shifts.

Comparison	Alpha Change	Theta Change	TAR Change	DAR Change	BAR Change	Largest Retained GC Change	SPEC Closest Centroid	GC Closest Centroid	GC + SPEC Closest Centroid
Eyes-open before stimulation	+5.04%	+33.19%	+22.73%	−48.50%	−17.93%	Broadband global max, +161.97%	FTD → AD	HC → HC	HC → HC
Eyes-closed before stimulation	+19.92%	+79.66%	+34.88%	−17.80%	−16.00%	Broadband central-to-temporal, +260.05%	HC → AD	HC → HC	HC → HC
Eyes-open after stimulation	+38.75%	+45.55%	−2.58%	−42.31%	−20.83%	Broadband central-to-parietal, −91.17%	FTD → FTD	HC → HC	HC → HC
Eyes-closed after stimulation	+5.31%	+61.97%	+42.33%	−23.72%	−14.09%	Broadband global max, +456.61%	HC → HC	HC → HC	HC → HC

**Table 10:** Comparison of selected EEG-based AD/FTD/HC classification studies and methodological considerations relevant to the present GC-based analysis. Not reported (NR) indicates that the item was not directly reported or was not comparable in the same form.

Study	Data Source	Preprocessing/Denoising	Features and Feature Selection	Classifier/Validation	Reported Performance	Main Methodological Consideration
Rostamikia et al. [2]	Same public resting-state EEG dataset with 36 AD, 23 FTD, and 29 HC subjects	EEG preprocessing and artifact handling were applied according to the original study pipeline.	Multiple discriminative EEG features from time-, frequency-, connectivity-, and complexity-related domains; statistical feature selection was used.	SVM classifier with tenfold cross-validation.	DEM vs. HC accuracy of 93.5%; AD vs. FTD accuracy of 87.8%. F1-score and permutation *p*-values were NR in directly comparable form.	Reported high classification accuracy, but the validation and feature-selection strategy differs from the present nested subject-level framework. GC-derived directed connectivity descriptors were not evaluated as the main feature family.
Lal et al. [13]	Same public resting-state EEG dataset with AD, FTD, and HC subjects	Band-pass filtering, artifact rejection, and epoch/window-based processing were applied before feature extraction.	Sliding-window feature extraction with high window overlap; SVD entropy was reported as the best-performing feature representation.	KNN classifier with cross-validation based on windowed EEG segments.	Approximately 91–93% accuracy/F1-score across AD vs. HC, FTD vs. HC, and AD vs. FTD tasks. Permutation *p*-values were NR.	Strong numerical performance was reported, but the result depends on the windowing and overlap strategy. The approach does not test whether GC-derived directed connectivity descriptors add complementary information to spectral slowing markers.
Cai et al. [7]	Same public resting-state EEG dataset with 36 AD, 23 FTD, and 29 HC subjects	Study-specific EEG preprocessing was applied before multidimensional feature extraction.	Spectral, nonlinear-dynamics, and graph-theoretical EEG measures; feature fusion and dimensionality-reduction strategies, including LASSO- and PCA-based approaches, were evaluated.	Machine-learning classification models were evaluated using cross-validation; performance was reported using accuracy and AUC.	Graph-theoretical measures achieved the highest AD vs. FTD classification accuracy of 81.36%. Permutation *p*-values were NR in directly comparable form.	Provides a multidimensional EEG framework, but focuses on spectral, nonlinear, and graph-theoretical descriptors rather than bivariate GC-derived predictive directed connectivity features.
Mlinarič et al. [8]	Same public resting-state EEG dataset with 36 AD, 23 FTD, and 29 HC subjects	Preprocessed data were filtered between 0.5 and 45 Hz; artifact subspace reconstruction, ICA-based artifact rejection, common-average reference, 5 s epoching, and bad-channel or bad-trial handling were used.	Multiple functional-connectivity matrices across EEG frequency bands; filter-based and wrapper-based feature selection were used in the ensemble framework.	Functional-connectivity classifiers with stacked ensemble learning and leave-one-subject-out cross-validation.	Stacked ensemble ROC-AUC of 81.80% for AD vs. HC, 71.36% for FTD vs. HC, and 65.10% for AD vs. FTD. F1-score and permutation *p*-values were NR in directly comparable form.	Uses a careful subject-level validation framework and shows that AD vs. FTD discrimination remains challenging. The study focuses on functional-connectivity metrics and manifold/ensemble-based classification rather than spectral ratios combined with GC-derived directed connectivity descriptors.
Present study	Same public resting-state EEG dataset with 36 AD, 23 FTD, and 29 HC subjects, with an exploratory external PBM case-study projection	EEGLAB-based preprocessing, average referencing, 0.5–45 Hz band-pass filtering, spectral noisy-channel screening, ICA/ICLabel-based artifact-component rejection, and post-artifact-removal average rereferencing.	Spectral power, TAR/DAR/BARs, and broadband and frequency-specific bivariate GC-derived directed connectivity descriptors. Feature ranking was performed inside training folds using Kruskal–Wallis tests for three-class classification and Wilcoxon rank-sum tests for binary classification. The number of retained features was selected in the inner CV loop.	Linear SVM ECOC for three-class classification and linear SVM for binary tasks; nested 5-fold outer/4-fold inner cross-validation; FDR correction and permutation testing.	Three-class spectral-only balanced accuracy of 0.572 and macro-F1 of 0.557. DEM vs. HC GC + SPEC balanced accuracy of 0.710 and macro-F1 of 0.665, with permutation testing significant at p<0.001. AD vs. FTD GC + SPEC achieved the highest numerical performance in the main nested-CV comparison, but the permutation test was not significant (p=0.270).	Provides a conservative and statistically transparent evaluation of spectral and GC-derived directed connectivity markers. Main limitations are the small and imbalanced dataset, scalp-level analysis, sensitivity of bivariate GC to common-source and volume-conduction effects, and limited statistical support for AD vs. FTD classification.

## Data Availability

The EEG data used in this study were obtained from a publicly available resting-state EEG dataset of subjects with AD, FTD, and HC, originally collected at the 2nd Department of Neurology, AHEPA General Hospital, Thessaloniki, Greece, and described by Ref. [15]. The PBM EEG data used in the exploratory case-study analysis were previously presented in [6].

## Abbreviations

The following abbreviations are used in this manuscript:

AD	Alzheimer’s disease
AIC	Akaike information criterion
AR	Autoregressive
BAR	Beta/alpha ratio
CV	Cross-validation
DAR	Delta/alpha ratio
DEM	Dementia group
EEG	Electroencephalography
FDR	False discovery rate
FTD	Frontotemporal dementia
GC	Granger causality
HC	Healthy controls
ICA	Independent component analysis
PBM	Photobiomodulation
SPEC	Spectral features
SVM	Support Vector Machine
TAR	Theta/alpha ratio
